# A Decision-Centric Benchmark of Enrichment and Potency Ranking in CNN-Rescored Molecular Docking

**DOI:** 10.3390/ijms27219652

**Published:** 2026-10-28

**Authors:** Yen-Hsun Lai, Hsiang Chi Huang

**Affiliations:** 1 Department of Chemistry, National Dong Hwa University, Hualien 974301, Taiwan; 2 MediSense Co., Ltd. (NaCo·AI), Taichung 404025, Taiwan

**Keywords:** molecular docking, scoring functions, virtual screening, enrichment vs. ranking, property-matched decoys, CNN rescoring, benchmark construction, pose prediction, analogue bias, negative result

## Abstract

Virtual screening answers two different questions with one number: **enrichment**, separating actives from background molecules, and **potency ranking**, ordering actives by measured activity. We benchmarked both for the same score on the same compounds across five CNS binding sites (SERT, GABA_A_ benzodiazepine site, 5-HT_1A_, σ_1_, and NMDA MK-801 pore), docking ChEMBL actives, inactives, and property-matched decoys with Uni-Dock and GNINA under a held-out normalisation/evaluation split, plus an experimentally characterised negative probe excluded from all fitting. Ranking, evaluated among pChEMBL ≥ 7 actives, was negligible for every target (median Spearman ρ = 0.020) and was not recovered by data-quality filtering, multivariate null models, scaffold-aware resampling, strict assay–site matching with rebuilt decoys, or stricter curation. Enrichment proved strongly benchmark-dependent: an apparent ΔAUC gain of +0.153 against molecular weight-matched decoys decreased to ≤0.05 once the decoys were matched to seven descriptors, and the residue was not robust to compound resampling. A classifier using no structural information separated the same actives from the same decoys at ROC-AUC 0.959–0.993, and cognate ligand redocking reproduced the crystallographic pose as the top-ranked solution for one of five targets. Benchmark enrichment should not be equated with protein-specific recognition; the benchmark, the poses, and the ranking require separate validation.

## 1. Introduction

Through structure-based virtual screening, it is possible to generate poses with a docking engine and rescore them, increasingly with machine-learned functions [1]. A persistent source of confusion is that one number is used to address two different issues. **Enrichment** measures whether a score separates known active compounds from background molecules assumed not to bind—a classification task, usually evaluated as ROC-AUC against property-matched decoys. **Ranking** measures whether the score orders known actives by measured potency—a correlation problem, and a much harder one. The distinction is operational: enrichment determines which compounds enter a shortlist, while ranking determines which of them is made next. A pipeline adequate for the first decision may be uninformative for the second, and a single aggregate figure of merit conceals the difference. This decision-centric framing—what a score lets a project actually achieve, rather than how well it correlates in the abstract—has recently been applied to screening triage under fixed budgets, where docking-first funnels recovered fewer actives than ligand-based prioritisation under a matched budget [2].

The contribution of this work is an evaluation framework rather than a scoring algorithm. We apply a reference-distribution-adjusted score (RDAS), defined as the z-score of each compound’s CNNaffinity relative to the property-matched null distribution within its molecular-weight bin. It aims to determine not how high a raw score is but how unusual it is relative to a size-matched background, so that a molecule being assigned a score simply due to being large is not mistaken for genuine excess. The construction of the framework is not new: z-scoring against a matched reference is standard, and we treat it as a representative instance of that family. Docking and CNN scores are known to favour larger and more lipophilic molecules irrespective of affinity, and correcting against property-matched decoys is the usual remedy [3]. What we test is whether such de-biasing changes *decision-relevant* performance—which compounds enter a shortlist, how they are ordered within it, and whether a known false-positive is demoted—rather than whether it shifts the score distribution, which it does by construction.

Existing strategies differ in what they correct and, crucially, in what they evaluate (Table 1). Decoy-based benchmarks construct property-matched decoys to measure enrichment but neither correct the score nor test potency ranking. Ligand efficiency normalisation [4] divides affinity by heavy-atom count, a heuristic ranking metric rather than a calibrated correction method. Score de-trending through property regression, in Δ-machine learning correction [5], removes a fitted offset but is rarely assessed for circularity or for any effect on ranking compared with experimental data.

Four sources of bias, detailed as follows, make such claims hard to evaluate, and this study is built around controlling them. **Benchmark construction**: Reported screening performance is sensitive to how the comparison set is built, since analogue and property bias in common decoy collections can produce enrichment reflecting gross physicochemical differences rather than pocket complementarity [3,6,7], and ligand-based benchmarks can reward chemotype memorisation over generalisation [8]. **Circularity**: If the correction is estimated and evaluated based on the same molecules, the evaluation is self-fulfilling. **Assay–site correspondence**: If the docked pocket is not the site that the reference assay measures, any correlation is uninterpretable, whatever the scoring function. **Range restriction**: Confining the actives to a narrow potency band attenuates rank correlation independently of the score’s quality.

Therefore, we benchmark five CNS binding sites and, for the same score on the same compounds, (1) separate enrichment from within-active potency ranking and report both; (2) evaluate the correction under an explicit normalisation/evaluation null split, so that no molecule used to estimate it is used to score it; (3) quantify how much apparent enrichment is attributable to decoy construction by rebuilding the decoys based on seven descriptors and redocking them under an identical protocol; (4) include an experimentally characterised negative probe never used to construct or tune anything; (5) require assay–site matching as a precondition for any score-versus-experiment comparison; and (6) consider whether de-biasing changes what a project can output. The practical question is not whether the RDAS is a good correction method but which decisions are supported by docking with CNN rescoring and which remain unsupported.

## 2. Results

### 2.1. The Correlation Between Ranking and Experimental Data Is Near-Zero, and Neither Correction nor Data-Quality Filtering Rescues It

**Figure 1:**
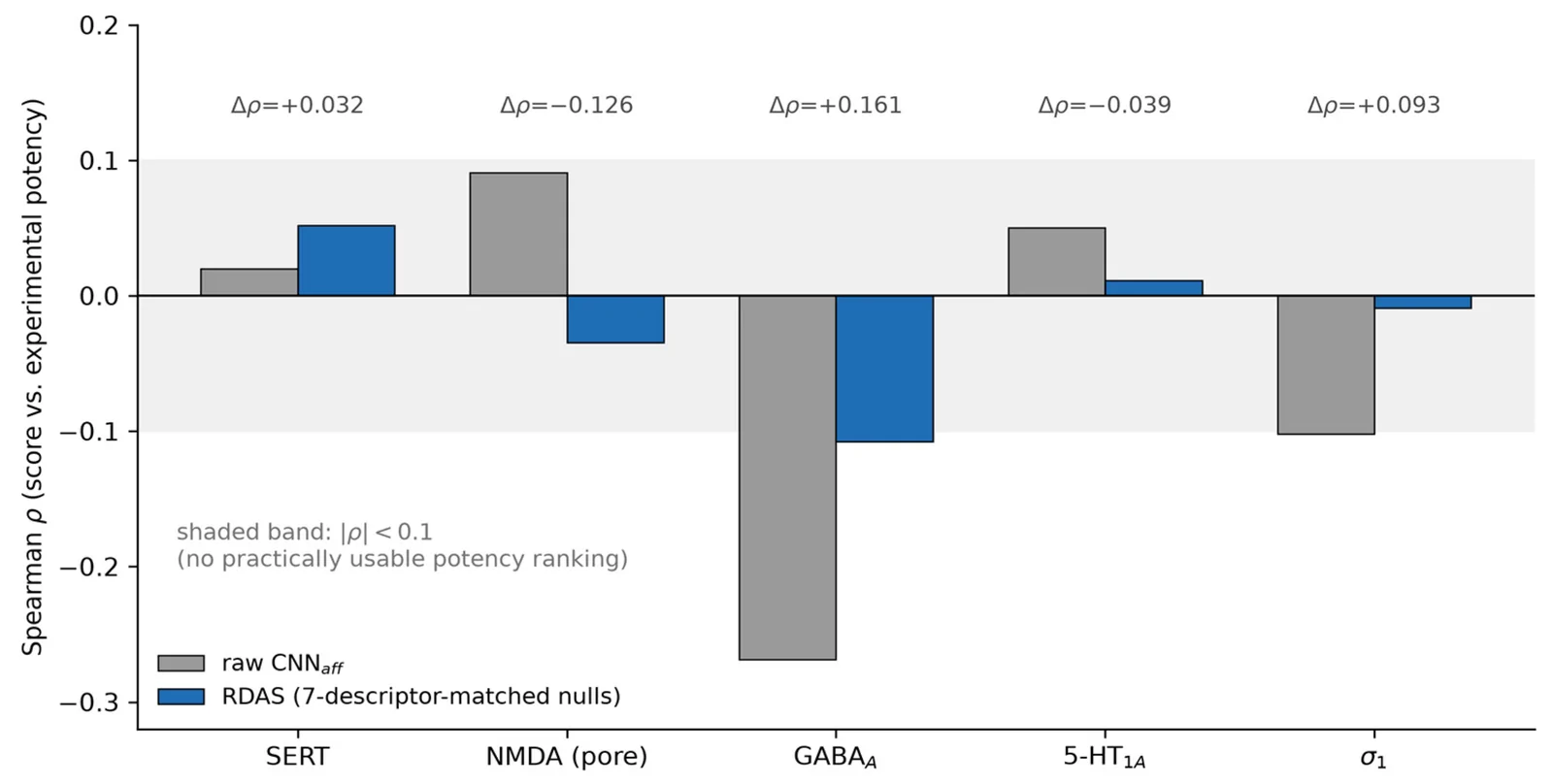
Rank correlation between GNINA CNNaffinity and experimental potency (Spearman ρ), over actives, for each target: raw versus property-matched-null-corrected score (RDAS). ρ is near-zero for every target under both scoring models; the shaded band marks |ρ| < 0.1, below which ranking is not practically usable. GABA_A_ and σ_1_ fall outside the band on the negative side. The correction does not produce usable ranking for any target (Δρ annotated; median Δρ = +0.032). Computed against the seven-descriptor-matched nulls, consistent with Table 2.

The Spearman correlation between score and experimental potency was negligible for every target, under both raw and RDAS scoring (Figure 1, Table 2). Critically, **no targets showed a significantly positive correlation**. According to the permutation test, three targets were statistically indistinguishable from zero (SERT: ρ = +0.02, *p* = 0.58; NMDA: +0.09, *p* = 0.20; 5-HT_1A_: +0.05, *p* = 0.16; bootstrap 95% CIs all spanning zero), and two were significantly **negative**, i.e., their score anti-correlated with potency: GABA_A_ (ρ = −0.27, *p* < 0.001, 95% CI [−0.38, −0.15]) and σ_1_ (ρ = −0.10, *p* = 0.004, [−0.17, −0.03]). The GABA_A_ comparison set is the one that needs qualifying the most: only 5.0% of its activity records are relative to the benzodiazepine site, and 84.8% are electrophysiological, uniquely so among the five targets (Table S3). Restricting to assay-defined subsets of the same docked compounds does **not** remove the anti-correlation; it is preserved and, if anything, stronger in the better-matched subsets (Table S4). However, 70% of the set derives from a single source, and the pooled estimate of −0.27 is partly amplified by a between-source offset, falling to −0.172 once that offset is removed. In Section 2.9.3, we present the stratification. The conclusion that matters here is unchanged: **no subsets, however curated, recovered useful positive ranking**.

The medians across targets were ρ = 0.020 (raw) and −0.009 (RDAS), and the RDAS did not produce usable ranking (median Δρ = +0.032). The same pattern held for Vina scores (ρ was weak or negative throughout), indicating that the failure is not specific to the CNN rescorer.

**Figure 2:**
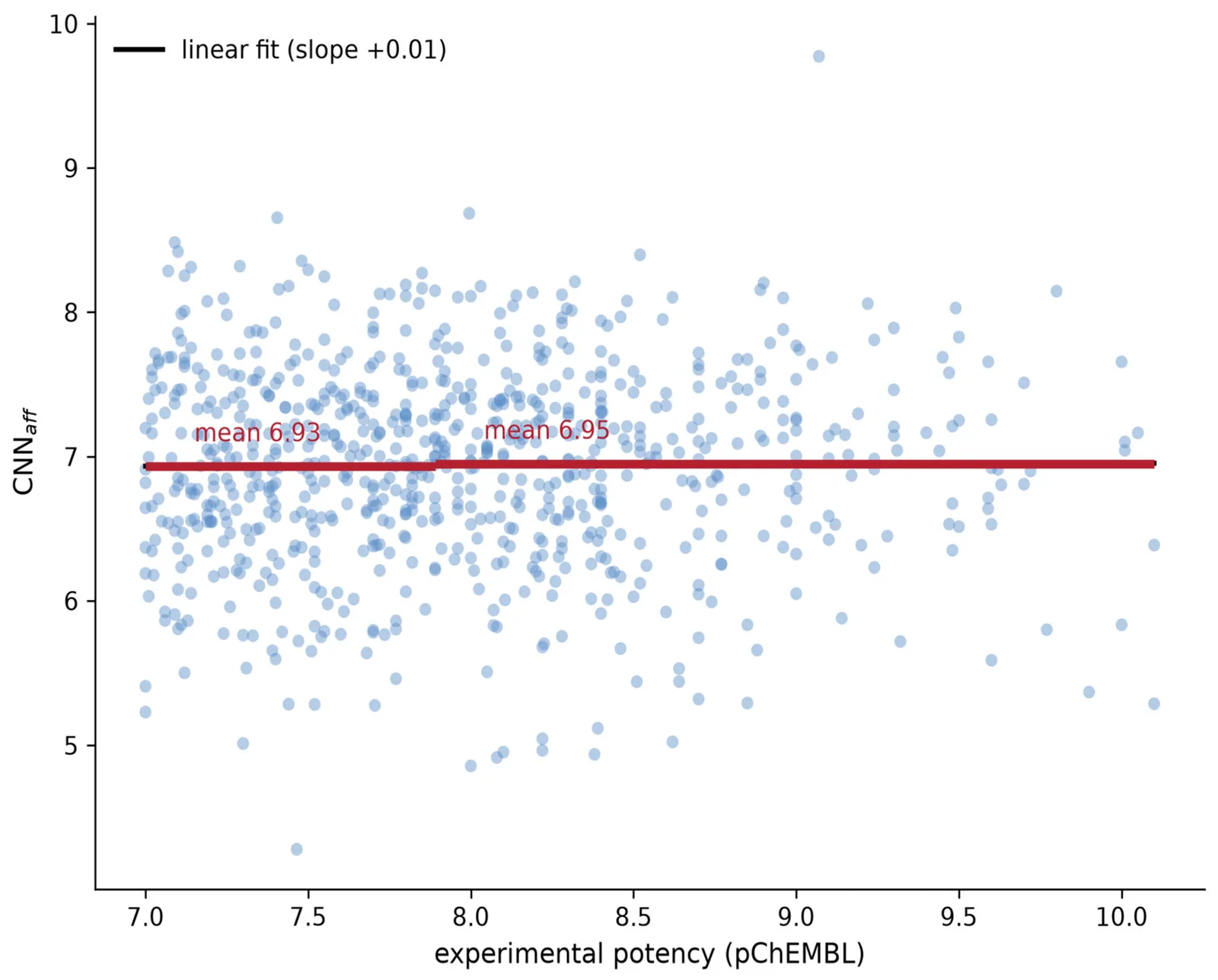
SERT actives (n = 793): CNNaffinity versus experimental potency. CNNaffinity spans 4.3–9.8 but shows no trend with potency (linear-fit slope: +0.01; Spearman ρ = 0.02, *p* = 0.58, n.s.). Red segments mark the mean CNNaffinity of the lower- and upper-potency halves (6.93 and 6.95), which are essentially identical—there are no within-active potency signals to recover.

**Figure 3:**
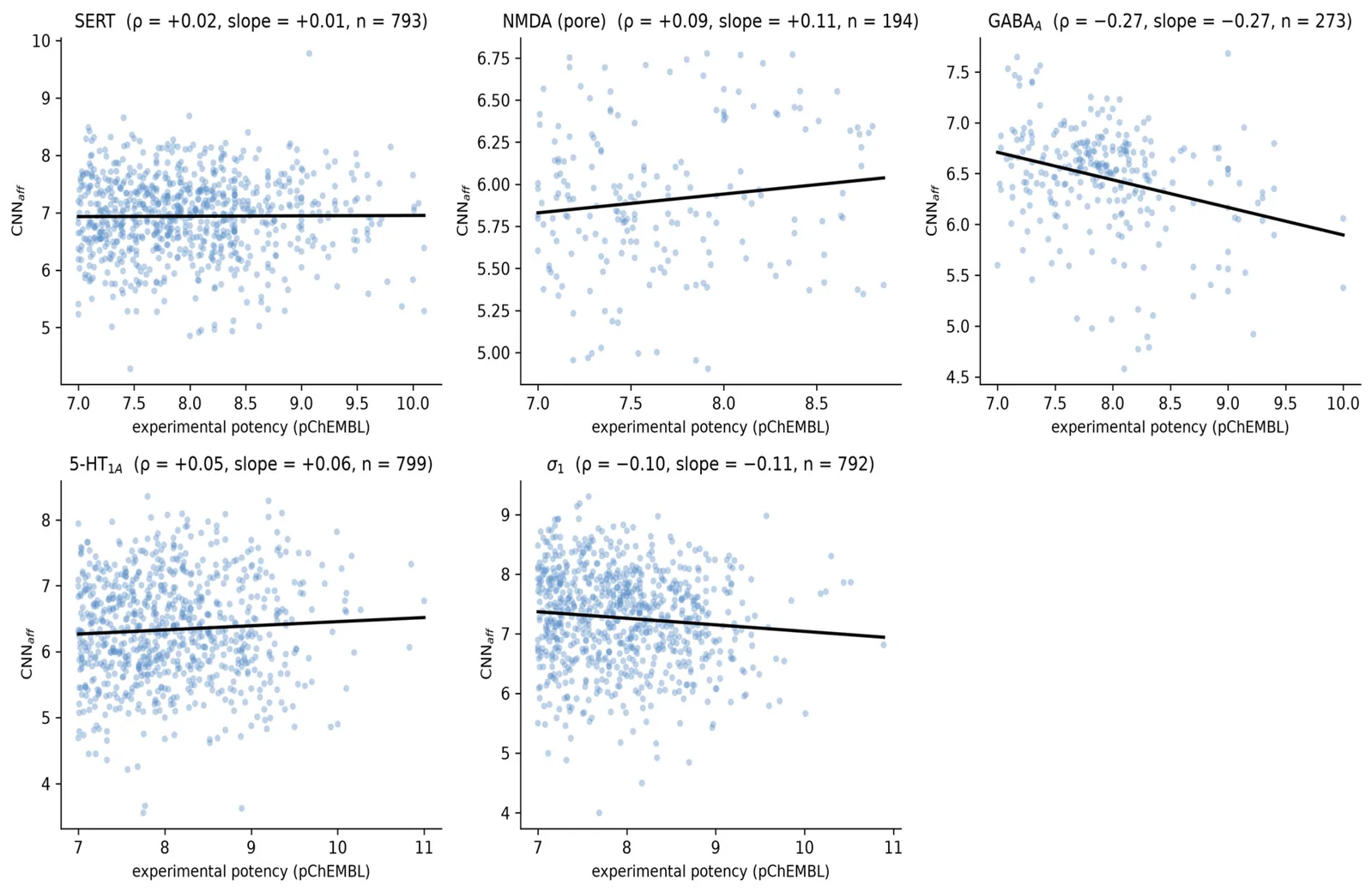
CNNaffinity versus experimental potency for actives for all five targets. Slopes are flat or negative throughout; GABA_A_ (ρ = −0.27) and σ_1_ (ρ = −0.10) trend the wrong way. No target shows useful positive potency ranking, which indicates that the result for SERT (Figure 2) is systemic.

A within-active diagnostic clarifies *why* ranking fails. For SERT, CNNaffinity spanned 4.3–9.8 across actives whose experimental potency spanned only 7.0–10.1 (a range-restricted, uniformly potent band); the mean CNNaffinity of the lowest- and highest-potency actives was essentially identical (6.93 vs. 6.95; Figure 2). The score, therefore, carries no monotonic potency information within the actives, so there are no signals for any correction to recover. The near-zero Δρ is the expected consequence, not a failure of the RDAS per se. The same pattern of flat or negative within-active slopes held across all five targets—no targets showed useful positive potency ranking, and GABA_A_ (ρ = −0.27) and σ_1_ (ρ = −0.10) trended slightly the wrong way (Figure 3), which indicates that the result for SERT is systemic rather than target-specific.

To clarify whether this near-zero correlation reflects the *model* or the *data*, we characterised the experimental side directly and repeated the ranking analysis on progressively cleaner subsets of the actives. The ChEMBL actives are severely range-restricted (87–100% of actives fall within a two-log-unit window, pChEMBL ∈ [7, 9]; the per-target interquartile range is 0.67–1.09 log units) and noisy (77–94% of actives derive from a single measurement; assay types are mixed K_i_/IC_50_/K_d_, as shown in Supplementary Table S5). When the actives are filtered by single-assay-type (Ki-only) and/or replicated (n_meas ≥ 2) measurement, ρ rises for two targets (SERT 0.020 → 0.147; 5-HT_1A_ 0.050 → 0.191) but remains unchanged or negative for the other three (GABA_A_ stays at −0.18 and σ_1_ at ≈0, and NMDA is unstable at small n; Table S5). The cleanest subsets are also the smallest (GABA_A_ n = 5; NMDA n = 13), so those estimates are not robust.

This is consistent with independent evidence showing that the apparent affinity-prediction performance of deep learning scoring functions is substantially inflated by training–test leakage in the standard benchmarks: removing structurally redundant complexes between PDBbind and CASF and retraining raises the error of state-of-the-art models considerably [9], which implies that the reported scoring power overstates what such models recover on actually unseen chemistry.

In conclusion, data quality limitations explain *at most a small part* of the failure: cleaning the data nudges ρ upward for some targets but never produces usable ranking (the largest clean-subset ρ across all five targets is 0.19, which is far below a level that would support reliable practical rank ordering), and for three of the five targets, it does not help at all. The appropriate claim is, therefore, scoped: *within the potency range and data quality attainable from ChEMBL for these targets, CNN-rescored docking—corrected or not—provides no usable potency ranking.* We do not claim that the score has zero latent ranking capacity in principle; we claim that no ranking that is useful in practice is recoverable here and that the limitation is removed neither by applying RDAS correction nor by restricting the data to the highest-quality experimental data.

**Table 2:** Test A—ranking and enrichment (GNINA CNNaffinity).

Target	n_Active	ρ (Raw)	ρ (RDAS)	Δρ	AUC Raw	AUC RDAS	ΔAUC [95% Split Interval]	Splits > 0
SERT	793	+0.020	+0.052	+0.032	0.658	0.680	+0.022 [+0.015, +0.031]	100%
NMDA (pore)	194	+0.091	−0.034	−0.126	0.569	0.613	+0.045 [+0.026, +0.063]	100%
GABA_A_	273	−0.268	−0.108	+0.161	0.730	0.742	+0.013 [−0.004, +0.027]	92%
5-HT_1A_	799	+0.050	+0.011	−0.039	0.570	0.567	−0.003 [−0.009, +0.002]	8%
σ_1_	792	−0.102	−0.009	+0.093	0.599	0.614	+0.014 [+0.003, +0.024]	100%
**Median**		**+0.020**	**−0.009**	**+0.032**			**+0.014**	

**Figure 4:**
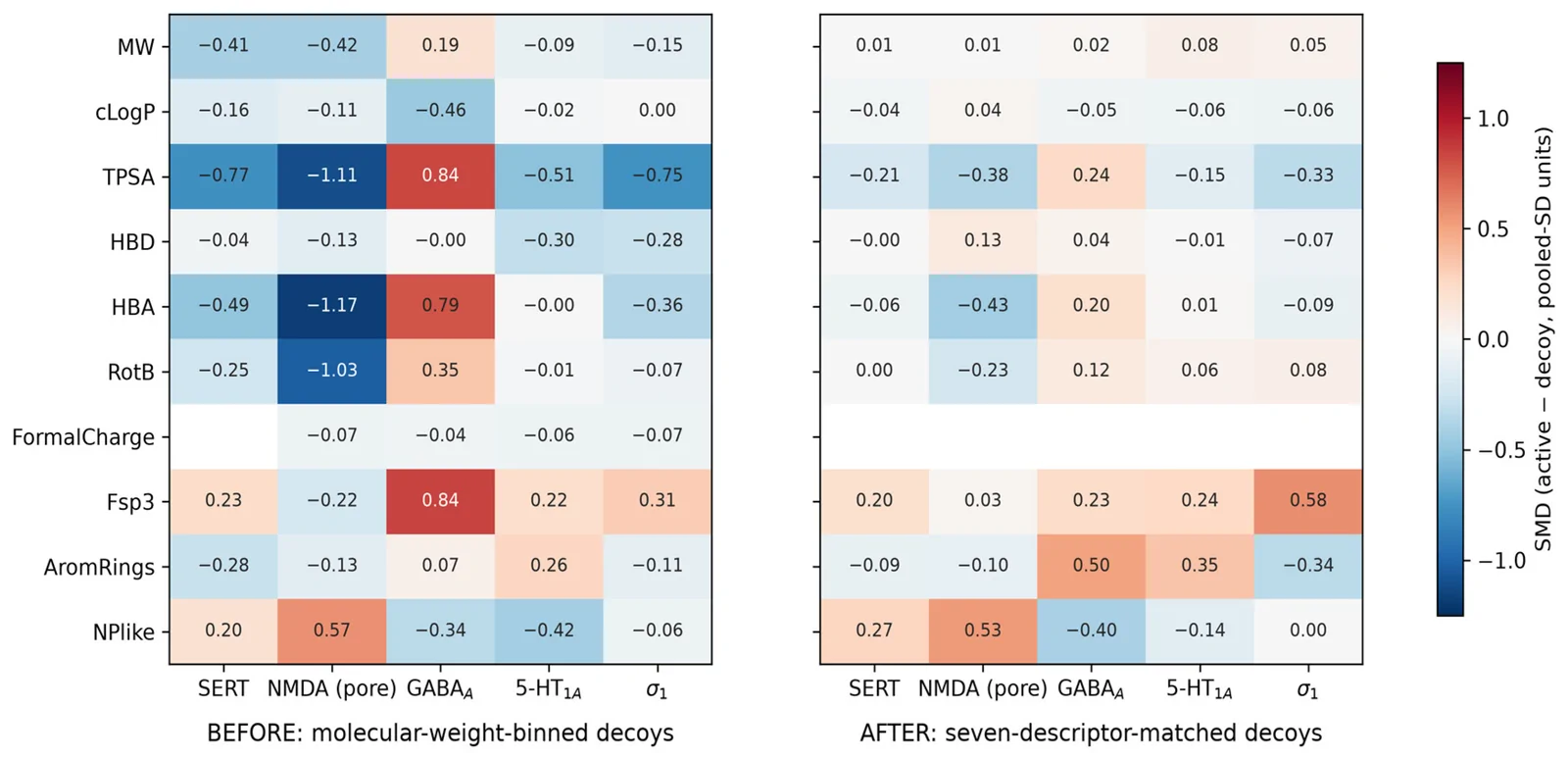
Null-matching audit: standardised mean difference (active–decoy, pooled-SD units) across ten descriptors for the initial **MW-binned** decoys (**left**) versus the redesigned **seven-descriptor-matched** decoys (**right**). Matching reduces |SMD| from ≈0.8–1.2 (TPSA, HBA, and RotB) to ≤0.44 on the matched descriptors; residual differences in Fsp^3^, aromatic rings, and NP-likeness remained on the descriptors that were not matching variables.

Ranking: ρ is near zero under both raw and corrected scores; no ρ is significantly positive, and GABA_A_ and σ_1_ are significantly **negative** (permutation test; see Section 2.1). Enrichment: AUC and ΔAUC are computed against the **seven-descriptor-matched** decoys and summarised over 200 independent random normalisation/evaluation partitions (mean, with the 2.5–97.5 percentile interval and the proportion of partitions favouring correction). ΔAUC is positive for SERT (+0.022), NMDA (+0.045), σ_1_ (+0.014), and GABA_A_ (+0.013) and negative for 5-HT_1A_ (−0.003); all |ΔAUC| ≤ 0.05. Bootstrap intervals over compounds and over Bemis–Murcko scaffold families are given alongside the split intervals in Table S14 and include zero for every target under scaffold-level resampling. The ΔAUC with reference to the earlier MW-binned decoys was three- to five-fold larger (up to +0.153); most of this difference is attributable to decoy design (Section 2.2, Figure 4, Table S9).

### 2.2. The Enrichment Gain Is Small, Mostly a Decoy-Matching Artifact, and Concentrated near the Top of the List, but It Is Inconsistent

Upon closer inspection, the enrichment result is reversed, which is a more useful finding. Against our **initial MW-binned decoys** and evaluated under the same held-out protocol, the correction appeared to improve the active-versus-decoy AUC substantially, with ΔAUC being +0.103 (SERT), +0.153 (NMDA), +0.055 (σ_1_), +0.022 (GABA_A_), and −0.019 (5-HT_1A_) (Supplementary Table S9). Taken at face value, this appeared to be a clear enrichment benefit.

A null-matching audit undercut the above reading. Computing the standardised mean difference (SMD) between actives and the MW-binned decoys on ten descriptors showed that although molecular weight was approximately controlled, the decoys were grossly mismatched in polarity and flexibility: |SMD| reached 1.11 (TPSA), 1.17 (H-bond acceptors), and 1.03 (rotatable bonds) (Figure 4, “before”). For four of the five targets, the CNS actives are more lipophilic and have lower polarity than a generic background, so a large part of the active-versus-decoy AUC—and its apparent improvement induced by the correction—reflected gross physicochemical class differences rather than pocket complementarity [6,7]. For GABA_A_, the imbalance ran in the opposite direction (TPSA SMD, +0.84).

**Figure 5:**
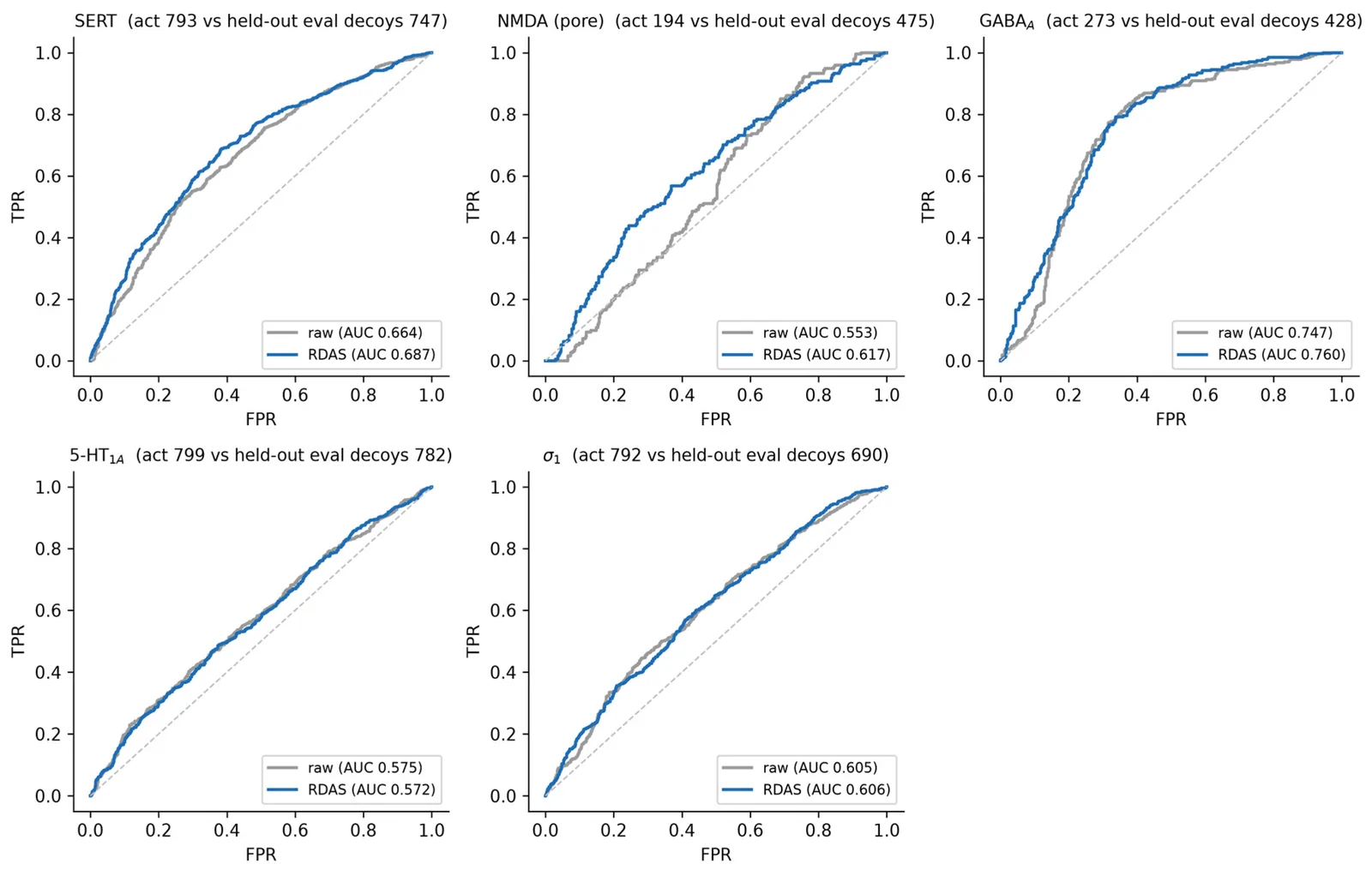
Enrichment ROC curves (actives versus **seven-descriptor property-matched decoys**) for each target—raw CNNaffinity versus RDAS. Curves are shown for one representative held-out normalisation/evaluation partition; summary statistics over 200 partitions are given in Table 2. Against matched decoys, the gain is small (ΔAUC ≤ 0.05), and absolute AUCs are modest (0.57–0.75). We compare with the larger apparent gain against MW-binned decoys (Section 2.2, Table S9).

Therefore, we rebuilt the decoys; matched per active on seven physicochemical descriptors (Section 4.2), which reduced the matched-descriptor SMDs to |SMD| ≤ 0.44 (Figure 4, “after”); and redocked them under the same protocol. Matching substantially reduced but did not eliminate physicochemical imbalance: residual differences persisted in fraction-sp^3^ carbon, aromatic-ring count, and NP-likeness, descriptors not used for matching. **Against the matched decoys, the apparent benefit largely collapsed** (Figure 5; Table 2): ΔAUC decreased to +0.022 (SERT), +0.045 (NMDA), +0.014 (σ_1_), and +0.013 (GABA_A_) and was absent in the case of 5-HT_1A_ (−0.003). The gain remained positive for SERT, NMDA, σ_1_, and GABA_A_ but was small (≤0.05) rather than within 0.10–0.15. For GABA_A_, the gain was smaller and less consistent than for the other improving targets (95% split interval [−0.004, +0.027]; positive in 92% of 200 random null splits).

These intervals answer a narrower question than they may appear to. The 200-partition interval is considered to determine whether an improvement is stable across the normalisation splits of a fixed decoy set; a bootstrap over **compounds** is considered to determine whether such an improvement would reappear in a new sample of molecules. Under the latter, the 95% interval included zero for three of the five targets (GABA_A_, 5-HT_1A_, and σ_1_), and under **scaffold-level** resampling (which treats a Bemis–Murcko family, rather than a molecule, as the sampling unit), it included zero for **all five** (Table S14). Therefore, we describe the RDAS as producing small, target-dependent shifts in decoy-benchmark enrichment that are stable across normalisation splits but **not robust to compound or scaffold resampling**, and we do not claim a significant improvement for any target. The absolute AUCs against the matched decoys are small throughout (0.57–0.73), which indicates that docking + CNN rescoring shows only modest discrimination on this constructed benchmark.

To express enrichment in terms closer to virtual-screening practice, we additionally report early-recognition metrics—enrichment factors at the top 1/5/10%, BEDROC (α = 20), and PR-AUC—alongside ROC-AUC (Supplementary Table S7). Three points emerge. First, the RDAS benefit is small and *target-variable*: the AUC improved for four of the five targets and BEDROC for another four, while EF at the top 1% improved for three (it decreased for GABA_A_, and for NMDA, the top 1% was effectively empty under both scoring models). Second, where the RDAS is helpful, this effect is *concentrated near the top of the list*: for SERT, it increased the top 1% from random (EF1% = 0.93) to 1.44 (ceiling ≈ 1.94 here), and across the targets, the BEDROC gains (e.g., NMDA, 0.17 → 0.32; GABA_A_, 0.49 → 0.63) generally exceeded the bulk AUC gains; i.e., the correction concentrates actives near the head of the list more than it shifts the overall AUC. Third, NMDA is the weakest target throughout—its top 1% contained essentially no actives under either scoring model (mean EF1%: 0.00 raw and 0.05 corrected). Enrichment factors, PR-AUC, and BEDROC all depend on class prevalence and must not be compared between sets with different decoy–active ratios: the attainable EF ceiling here ranges from 1.87 to 3.45 (Table S7). Cross-target comparison should, therefore, use AUC, which is prevalence-robust. Where a subset analysis changes the ratio (as the site-matched subsets of Section 2.9.3 do, from 1.88 to 3.76 for SERT and from 3.13 to 19.5 for GABA_A_), we downsample the evaluation decoys to the original ratio and average over 200 draws before reporting any early-recognition metric (Table S15); unmatched comparisons are not reported.

The mechanism is straightforward: the RDAS is an MW-binned de-trending method, so once the decoys are themselves MW- and property-matched, there is little residual bias for it to remove. **The large enrichment gain seen against decoys matched by molecular weight alone was, therefore, largely attributable to decoy design rather than to a property of the correction.** This is a concrete warning: the apparent benefits of score correction methods must be evaluated against decoys whose property match is demonstrated quantitatively, or they will be overstated.

Discrimination against experimentally labelled ChEMBL inactives behaved differently. Raw CNNaffinity resulted in AUCs of 0.675 (SERT), 0.739 (NMDA), 0.647 (5-HT_1A_), and 0.624 (σ_1_); GABA_A_ was not evaluable (n = 2 inactives). The correction did not improve discrimination in this comparison for any target and slightly reduced it (0.673, 0.704, 0.631, and 0.594, respectively; Supplementary Table S10). The enrichment benefit reported above is, therefore, specific to discrimination against property-matched background molecules and did not transfer to the smaller sets of experimentally measured inactives available here.

### 2.3. The Rank of an Experimentally Characterised Negative Probe Is Decreased for Every Site but Rarely Flagged

**Figure 6:**
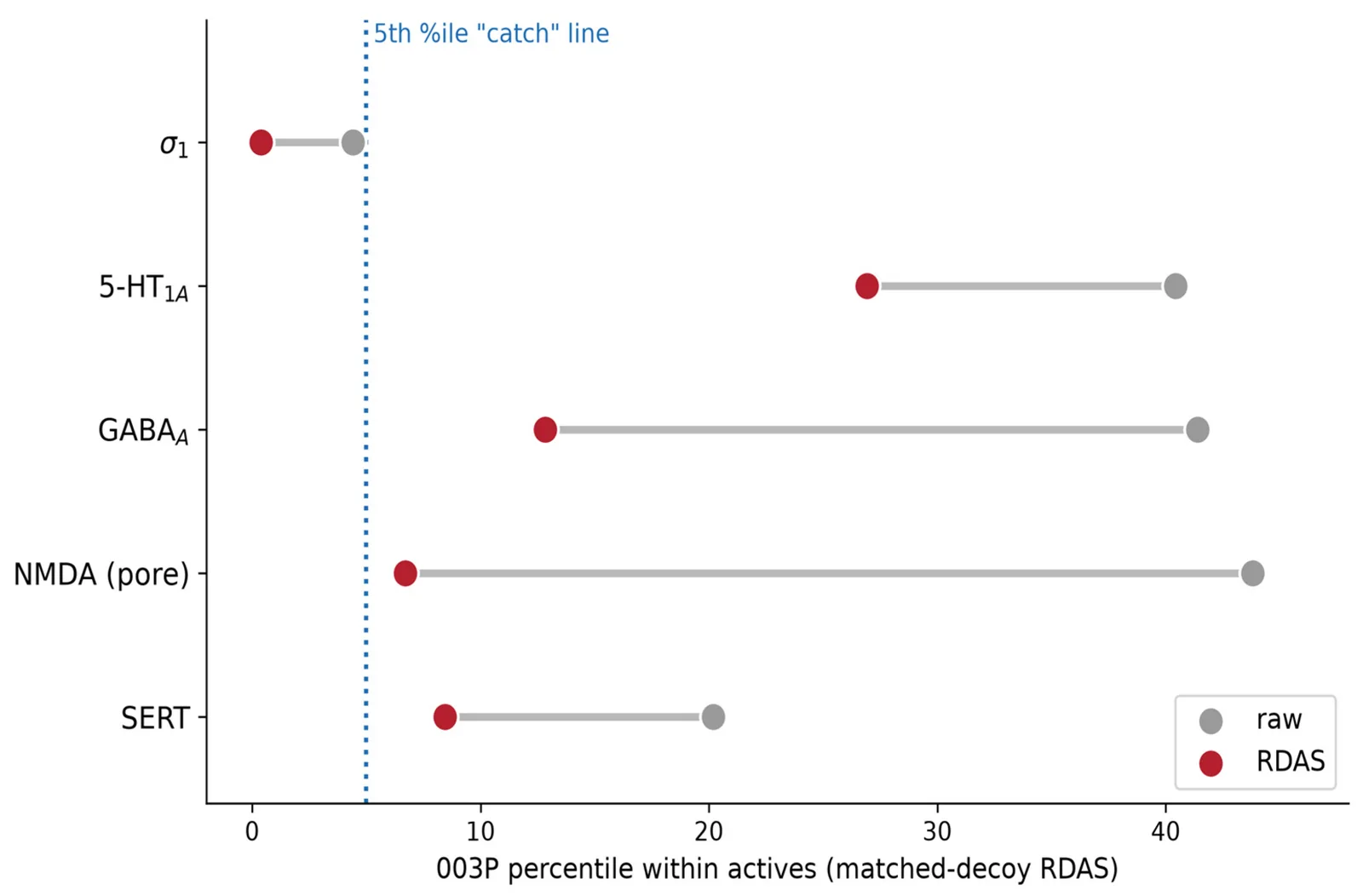
Within-active percentile of the experimentally characterised negative probe TFE-Rhy for each target, raw versus RDAS scoring (matched decoys). The RDAS shifts TFE-Rhy toward the inactive tail for all five targets (raw 4–44th → 0.4–27th), but it only crosses the fifth-percentile “catch” line for one of them (σ_1_).

Under the tested conditions and raw CNNaffinity, the negative probe TFE-Rhy ranked in the middle tier among the actives for three of the five targets (40th–44th percentiles for NMDA, GABA_A_, and 5-HT_1A_); i.e., it “looked active-like.” The RDAS lowered its within-active percentile for **all five** targets (raw 4–44th → RDAS 0.4–27th; Figure 6, Table 3). However, under the strict criterion of falling below the fifth percentile of actives, TFE-Rhy was “caught” at only **one** of the five targets (σ_1_), under both raw and RDAS scoring. The rank of the parent alkaloid, RHY, was likewise decreased under the RDAS model for all five targets.

De-biasing, therefore, reduces but does not eliminate false-positives: the realistic benefit is a percentile shift toward the inactive tail, not hard reclassification.

### 2.4. The Correction Is Not a Uniform Size Penalty

**Figure 7:**
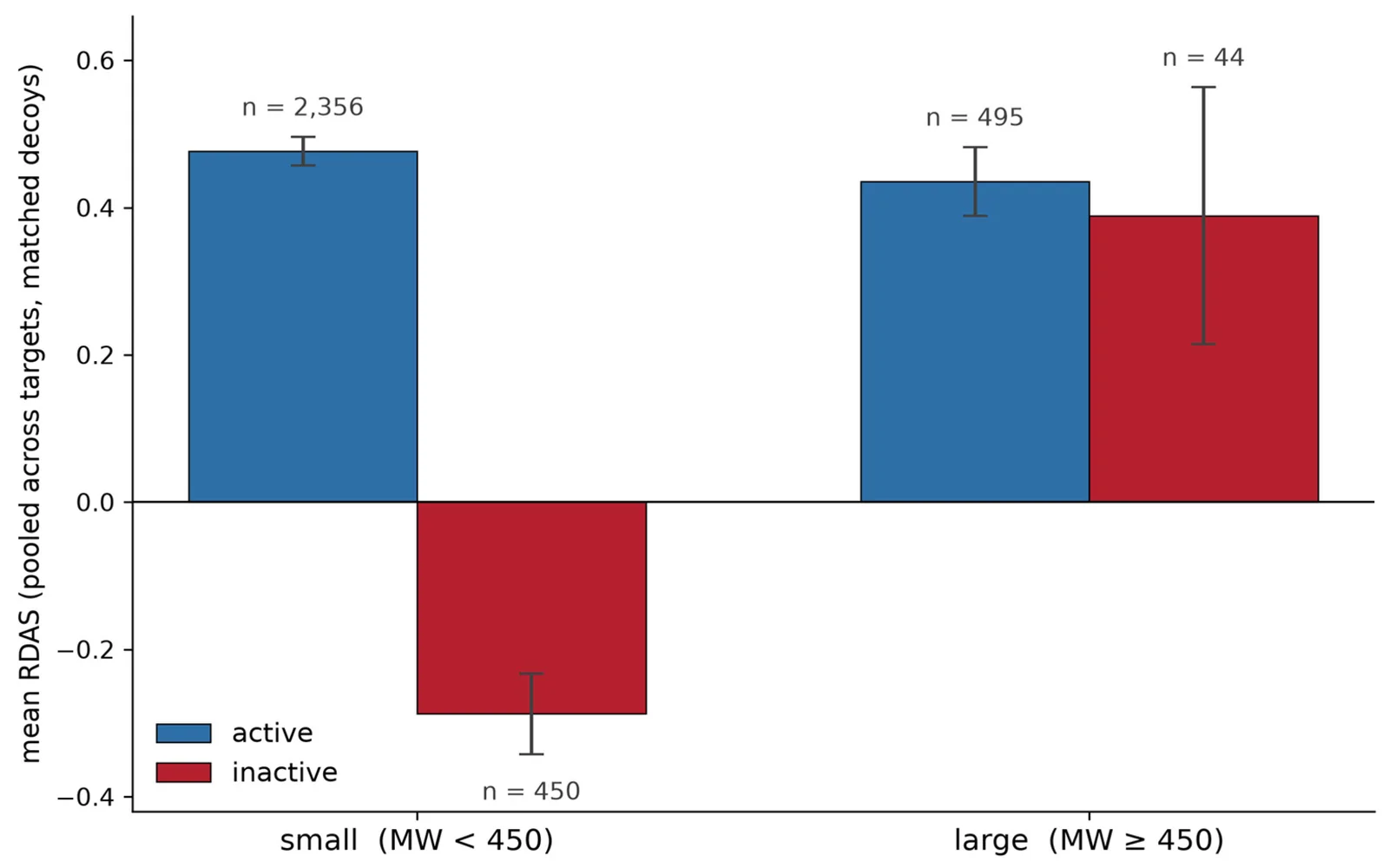
Mean RDAS (pooled across all five targets) stratified by ligand size (MW < 450 vs. ≥450) × activity; error bars are ± 1 standard error of the mean. Large actives retain a positive mean RDAS, which indicates that the correction is not a uniform size penalty, whereas active/inactive separation is visibly reduced in the large-molecule range. Pooling raises the large-inactive count from n = 1–2 per target to n = 44. The pooled analysis is reported descriptively, because compounds may occur at more than one target and the independence assumption required in a pooled rank test does not hold.

A concern with any size-based correction is that it merely penalises large molecules indiscriminately. It does not. When we pooled all five targets (which raises the otherwise sparse large-MW inactive count from n = 1–2 per target to n = 44; Figure 7), large actives retained a clearly **positive** mean RDAS (+0.44), confirming that the RDAS is not a uniform size penalty. However, the pooled analysis also refines the original claim: while the correction clearly separates actives from inactives among **small** molecules (mean RDAS: +0.48 vs. −0.29), the two groups are indistinguishable among **large** molecules (+0.44 vs. +0.39). We report this stratification descriptively and do not attach a significance test to it: compounds may appear at more than one target in the pooled set, so the independence assumption required in a pooled rank test does not hold. The discrimination that the RDAS provides, therefore, degrades precisely in the large, flexible range—consistently with the over-prediction of the large negative TFE-Rhy and with this study’s broader theme that gross-property confounding is the strongest for large NP-like chemistry (computed against the matched decoys; the conclusion is unchanged from the MW-binned decoys).

### 2.5. Assay–Site Matching Is Crucial (NMDA Case Study)

NMDA illustrates a failure mode that can silently invalidate a benchmark. The [^3^H]MK-801 assay measures the open-channel (PCP) site within the transmembrane pore. The parent alkaloid’s documented pharmacology—non-competitive, voltage-independent NMDA antagonism without interaction at the polyamine, Zn^2+^, proton, or redox sites [10]—excludes several modulatory sites but does not localise the compound to any particular pocket; an amino-terminal-domain (ATD)/allosteric mechanism is, therefore, a hypothesis rather than an established assignment. Therefore, three distinct NMDA sites were considered: an allosteric PAM pocket (the engine’s original default), the ATD/ifenprodil site (a hypothesis-driven allosteric control site), and the channel pore (the assay).

Docking TFE-Rhy at the **assay-matched** pore (5UN1) yielded higher CNNaffinity (5.81) than docking it at the ATD/ifenprodil site (3QEL; 4.34) and placed it among the pore actives as “active-like” (43.8th percentile, raw). The NMDA result, therefore, represents a false-positive ranking at the assay-matched site rather than a site selection artefact; under the RDAS model (against the matched decoys), then, the rank was decreased to the 6.7th percentile. Pore poses were highly reproducible across the docking seeds (median pairwise RMSD: 0.33 Å; 9/10 ligands < 2 Å). **Such reproducibility is not, however, evidence that the poses are correct.** The cognate ligand redocking of MK-801 into the same pore (Section 2.9.2) returned a median top-1 RMSD of 5.17 Å from the crystallographic mode, with an interquartile range of only 5.14–5.18 Å across fifty seeds: the solution is reproducible and consistently displaced. Reproducibility across docking seeds is not evidence of pose correctness, and a seed-stability check should never be reported as pose validation.

The general lesson (Box 1) is that the computational site must be matched to the experimental radioligand site before any score-versus-experiment comparison is meaningful.

Box 1 **Assay–site matching prevents meaningless benchmarking.**

Three candidate NMDA sites were considered for the same compound: (i) the engine’s default allosteric PAM pocket; (ii) a hypothesis-driven allosteric control site, the ATD/ifenprodil pocket (3QEL); and (iii) the assay-matched MK-801 channel pore (5UN1)—the site that [^3^H]MK-801 binding actually measures. TFE-Rhy scored the highest at the assay-matched pore, so its false-positive signal arose from the assay-matched site rather than from use of the wrong pocket. **The correct site can still produce a false-positive; but without site matching, the benchmark itself is uninterpretable**.

### 2.6. There Is No Size-Correlated Rescoring Attrition in the Analysed Data

Several of our analyses stratify by molecular size, so a size-dependent failure in CNN rescoring would bias them: if large ligands were preferentially dropped, the surviving large-ligand population would be a selected subset, and the size-stratified comparisons of Section 2.4 and Section 2.7 would not be interpretable. We therefore checked the failure rate directly. On the production hardware (NVIDIA A100-PCIE-40GB; NVIDIA Corporation, Santa Clara, CA, USA), GNINA rescoring failed for no ligand at any molecular weight: 0 of 10,130 receptor–ligand pairs, including all 1948 ligands above an MW of 500 (Table 4). Every compound that was docked was therefore also scored, and the size-stratified analyses reported here are not affected by size-correlated attrition.

### 2.7. Richer Null Models Improve Enrichment but Still Do Not Rescue Ranking

A natural objection to a molecular-weight-binned correction method is that it is too simple: perhaps a multivariate property model would both enrich better *and* recover ranking. We tested this with two additional, circularity-free correction techniques fit on the normalisation half of the matched decoys and applied to the actives and the held-out decoy half: a linear residual model with molecular weight, cLogP, and TPSA and a random forest residual model with six descriptors (MW, cLogP, TPSA, HBD, HBA, and rotatable bonds). The bias that these correction techniques target is real and not dominated by size alone: in a standardised regression of CNNaffinity on descriptors over pooled decoys, the largest coefficients were molecular weight (0.34), cLogP (0.16), and TPSA (0.08), with the remaining descriptors being much smaller (Supplementary Table S8); therefore, the MW-binned RDAS leaves residual lipophilicity- and polarity-driven bias uncorrected.

The two axes respond very differently to a richer correction (Table S8). For *enrichment*, the multivariate models outperformed the MW-binned RDAS for most targets. BEDROC rose relative to raw CNNaffinity for every target and relative to the molecular-weight-binned correction for four of five of them, the exception being the linear residual for GABA_A_ (e.g., NMDA, 0.32 → 0.38; GABA_A_, 0.63 → 0.68; SERT, 0.74 → 0.78 under the random forest residual), and ROC-AUC rose for every target under both residual models relative to the binned correction. For *ranking*, no correction helped: across MW-binned, multivariate-linear, and random forest residuals, every ρ remained within |ρ| < 0.13. None approached usable ranking, and the residual models moved the two anti-correlated targets (GABA_A_ and σ_1_) close to zero; none produced a positive correlation of practical magnitude. The separation between the two axes is, therefore, not an artefact of using a simple correction method; enrichment can be improved with a better null model, whereas potency ranking is uncorrectable with any of the null models tested. These results show that multivariate de-biasing can alter enrichment on this benchmark, but they do not establish protein-specific screening performance; the utility of both the docking score and the correction should be validated in the intended screening context. The failure to recover useful potency ranking persisted across all correction models tested. It is also consistent with reports that benchmark scoring power for deep learning affinity models falls substantially once training–test redundancy is removed [9].

### 2.8. The Enrichment/Ranking Separation Is Not an Artifact of the Score Head Chosen

GNINA produces a CNN pose score (CNNscore) alongside CNNaffinity, and their product (CNN_VS) is the composite intended for virtual-screening triage [11]. Because our analysis used CNNaffinity throughout, we repeated the three decision-relevant analyses—enrichment, within-active potency ranking, and the TFE-Rhy false-positive probe—for CNNscore and CNN_VS, using the existing GNINA output under the identical held-out null protocol (Supplementary Table S11).

Regarding **enrichment**, the composite was not uniformly better. CNN_VS improved the ROC-AUC over CNNaffinity only for SERT (0.694 vs. 0.658) and was lower for the other four targets, markedly so for GABA_A_ (0.653 vs. 0.730). For 5-HT_1A_, both CNNscore (0.460 [0.446, 0.476]) and CNN_VS (0.476 [0.461, 0.491]) fell below random discrimination against the matched decoys. Early recognition behaved differently from bulk AUC: CNN_VS resulted in a higher BEDROC than CNNaffinity for three of the five targets, and CNNscore resulted in the single strongest early-recognition result in this study for SERT (BEDROC, 0.937 [0.925, 0.947] versus 0.687 for CNNaffinity) while being weaker for 5-HT_1A_ and σ_1_. Applying the molecular-weight-binned correction to CNNscore or CNN_VS generally did not help and, in the case of SERT, substantially reduced early recognition (CNNscore, BEDROC 0.937 → 0.782); this is consistent with CNNscore being a bounded pose probability that does not support the same size-dependent offset that the correction is designed to remove.

Regarding **potency ranking**, no score head recovered usable signal. None of the thirty combinations across all three scores, both raw and corrected, and all five targets produced a positive Spearman ρ of 0.10 or greater; for CNNscore and CNN_VS, the largest absolute correlation was −0.175. The composite score, therefore, behaves as CNNaffinity does on this axis.

Regarding the **negative probe**, CNN_VS gave better false-positive control for NMDA (TFE-Rhy percentile: 43.8 → 6.7) and σ_1_ (4.4 → 0.0) but worse control for GABA_A_ (41.4 → 81.7) and 5-HT_1A_ (40.4 → 70.3). Under the fifth-percentile criterion, CNN_VS flagged TFE-Rhy at one of the five targets, the same as CNNaffinity; raw CNNscore flagged it at two.

Taken together, the separation reported in Section 2.1 and Section 2.2 is a property of this CNN-rescoring family rather than of the particular head analysed. Choosing the virtual-screening composite changes which targets enrich well and can sharpen early recognition at some, but it does not create within-active potency information, and it does not give consistent false-positive control on the one experimentally characterised negative probe available to us.

### 2.9. Robustness Analyses Do Not Alter the Main Interpretation

Six control analyses were added to determine whether the results above are properties of the scoring function or of the benchmark built around it. Table 5 summarises them; the full statistics, per-target values, and algorithmic detail are in Tables S14–S19, S21 and S23. Two of the six support findings are consequential enough to state in the main text and are elaborated upon below.

#### 2.9.1. The Benchmark Is Largely Solvable Without Structure

A classifier using only two-dimensional ligand features reached ROC-AUC values of 0.959–0.993 under five-fold cross-validation grouped on a Bemis–Murcko scaffold (Table S16), far above the 0.57–0.73 obtained by docking with CNN rescoring on the same task. The physicochemical descriptors alone reached 0.706–0.900; the difference is due to the fingerprint.

**Figure 8:**
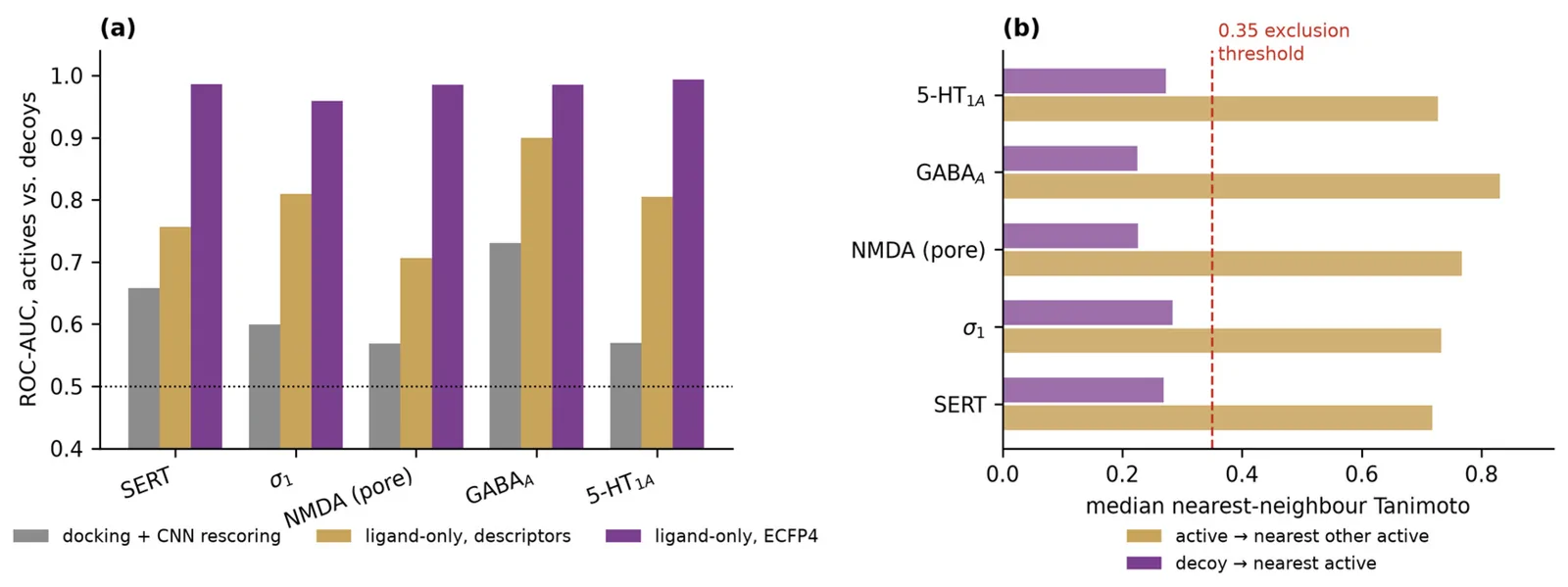
A structure-free classifier solves the enrichment benchmark and why. (**a**) ROC-AUC for separating actives from the property-matched decoys: docking with CNN rescoring (grey), a ligand-only model using twelve physicochemical descriptors (gold), and a ligand-only model using ECFP4 fingerprints (purple). All ligand-only values are from five-fold cross-validation grouped on Bemis–Murcko scaffold. (**b**) Median nearest-neighbour Tanimoto similarity: each decoy to its closest active (purple) and each active to its closest *other* active (gold). The dashed line marks the 0.35 analogue exclusion threshold applied when the decoys were selected, which caps every decoy below it while leaving the actives clustered well above it.

The mechanism follows from a documented design choice. Decoys were excluded if their Tanimoto similarity to any active reached 0.35, so every decoy’s nearest active sits at or below that threshold (medians, 0.225–0.284; maximum, 0.349 for every target), while 96.6–99.0% of actives have an analogue at or above 0.35 *within the active set* (nearest-neighbour medians, 0.718–0.831). The two classes, therefore, occupy disjoint regions of the fingerprint space **by construction**, and a fingerprint model need only determine whether a molecule has a close neighbour among the actives (Figure 8).

This is not a failure of property matching: only three of thirty target–descriptor pairs exceed |SMD| = 0.25 across the seven matching descriptors. The lesson is narrower and more useful. *Property matching does not guarantee a challenging decoy benchmark when active analogues are internally clustered but decoys are excluded from the same topological neighbourhood.* The absolute enrichment values reported here, therefore, cannot be read as evidence of protein-specific recognition, and ΔAUC is interpretable only as a within-pipeline comparison on a fixed constructed benchmark. The within-active ranking result is unaffected, being computed among actives only.

#### 2.9.2. Pose Generation and Pose Selection Are Separate Bottlenecks

The default seed of Uni-Dock is drawn from the clock, and two consecutive runs of the same ligand differed by 8.47 Å and 1.36 Å, so every cognate ligand was redocked under 50 explicit seeds; the per-target estimates settle by roughly 25 seeds (Table S19).

**Figure 9:**
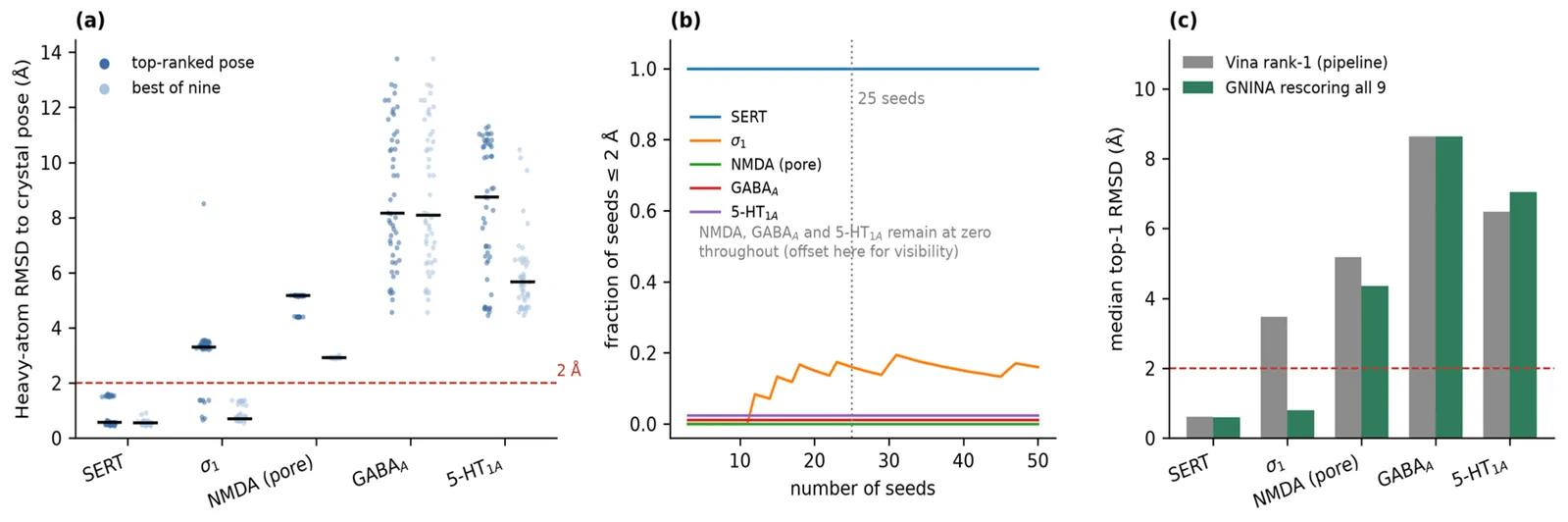
Cognate ligand redocking. (**a**) Heavy-atom RMSD from the crystallographic pose for the top-ranked pose (dark) and the best of nine poses (light) over fifty explicit seeds per target; horizontal bars are medians, and the dashed line is the conventional 2 Å criterion. (**b**) The fraction of seeds within 2 Å as a function of the number of seeds used, showing that the estimates settle by roughly 25 seeds. (**c**) Median top-1 RMSD when the pipeline takes the Vina rank-1 pose (grey) versus when the complete 9-pose list is rescored with GNINA (green); only σ_1_ is rescued, because at the other failing targets, the correct pose was never generated.

**Only SERT reliably recovered the crystallographic binding mode as the top-ranked pose across seeds** (median, 0.57 Å; 100% of seeds ≤ 2 Å; all three principal contacts re-formed). σ_1_ sampled the correct pose reliably (best-of-nine median, 0.70 Å; 100% of seeds within 2 Å) but ranked it below an incorrect pose. NMDA (5.17 Å), 5-HT_1A_ (8.74 Å), and GABA_A_ (8.16 Å) failed at both stages; for GABA_A_, all nine poses lay beyond 6 Å, so the correct mode was never generated (Figure 9).

**RMSD alone understates what is preserved.** The three residues in closest contact with each crystallographic ligand are re-formed far more often than the poses are: all three in 100% of seeds for SERT and σ_1_—the latter despite a median RMSD of 3.31 Å—and the canonical Asp116 salt bridge in 72% of seeds for 5-HT_1A_ at 8.74 Å (Table S23). A large RMSD for these targets more often means that a ligand anchored correctly and oriented wrongly rather than one that has missed the pharmacophore. Gross pocket disruption during receptor preparation is unlikely to be the cause, since each crystallographic ligand retains 11–15 residue contacts in the prepared receptor, although protonation, water, and cofactor choices may contribute. For every target, the crystallographic pose scored higher than the median of the production-selected poses, which is consistent with CNNaffinity being pose-sensitive.

Because the pipeline passes only the Vina rank-1 pose to GNINA, we tested whether the failure lies in selection. Rescoring the complete nine-pose list raised top-ranked recovery **from one target to two at no additional sampling cost**: for σ_1_, the correct pose obtained a Vina rank 4 and a GNINA rank 1 in 10 of 10 seeds. It rescued no other target and could not have—GNINA cannot re-rank a pose that was never generated. **Pose generation and pose selection are separate bottlenecks**, and only the second one can be repaired by rescoring poses already available.

#### 2.9.3. The GABA_A_ Active Set Is Dominated by a Single Assay

Of the 273 GABA_A_ actives, the **potency of 190 (70%) is derived from one ChEMBL assay, CHEMBL5737147** (193 molecules are annotated to that assay in the retrieved records, but 3 failed ligand preparation and so never entered the scored population, as shown in Table 6 and Table S20). The description of this essay indicates two-electrode voltage-clamp electrophysiology, although it is annotated as a binding assay, and the reported types are K_i_ with k_on_ and k_off_. Molecules from that source have systematically higher CNNaffinity and lower potency than the rest, a contrast that produces a negative correlation in the pooled data irrespective of any within-source relationship. Within the dominant source, ρ = −0.123 (*p* = 0.093); within the remaining 83 molecules, ρ = −0.283 (*p* = 0.0088); after rank-centring within the source, the pooled estimate decreases from −0.268 to −0.172 (Table S18). No single molecule drives it: the largest leave-one-out influence is 0.012.

Therefore, GABA_A_ should not be presented as the strongest evidence of anti-correlation. Importantly, **no source-stratified analysis recovered useful positive potency ranking**.

### 2.10. Aggregate Conclusions Are Stable Under the Stochasticity of the Docking Search

Because the seed of Uni-Dock was not fixed in the production run, a representative subset of each target (120 actives and 240 decoys) was redocked under 5 explicit seeds.

Per-molecule scores are not reproducible: the CNNaffinity of a single compound spans a median of 0.10–0.72 units across seeds and up to 3.62 units at worst. Between-seed rank correlation ranges from 0.626 (5-HT_1A_) to 0.927 (σ_1_). Therefore, 5-HT_1A_ is the least stable target throughout—and it is also the only target with a negative ΔAUC, which may not be coincidental.

The aggregates that support the conclusions are nevertheless stable. The raw enrichment AUC varies across seeds with a standard deviation of 0.007–0.022, far below the between-target spread. The sign of ρ is consistent across seeds for SERT, GABA_A_, and 5-HT_1A_; it changes sign for NMDA and σ_1_, with ρ being close to zero in this subset (+0.028 and −0.024). Sign instability is what the absence of signal predicts.

The comparison with the original run uses **archived production scores for identical sampled compounds**. The archived AUC falls inside the five-seed range for 5-HT_1A_ and NMDA and outside it by 0.007–0.008 for GABA_A_, SERT, and σ_1_. With only five seeds, the envelope is narrow, and small package-version differences between the original run and the revision environment cannot be excluded; therefore, we do not claim that the production run was a typical draw. All workflows added during revision set the docking seed explicitly.

## 3. Discussion

**Enrichment and ranking are separate, and de-biasing acts—weakly—only on enrichment.** Property-matched null correction removes a score’s systematic, property-dependent offset, and against decoys matched by molecular weight alone, it appeared to deliver a large enrichment gain. Once the decoys were themselves matched to seven descriptors, most of that benefit disappeared (Section 2.2); what remained was stable across normalisation splits but not robust to compound or scaffold resampling (Section 2.9). The correction cannot create a potency signal that the underlying score does not contain, and the SERT diagnostic shows why (Figure 2): CNNaffinity is flat across the actives’ potency range, so there is nothing to rank, and Δρ ≈ 0 follows necessarily. A de-biasing step may alter benchmark enrichment modestly, but such changes should not be interpreted as evidence of protein-specific screening performance without validation under an application-relevant library.

**Benchmark construction changes the apparent answer.** Our own first-pass decoys made the correction look three- to five-fold more effective than it is because CNS actives differ from a generic background in polarity and flexibility. Correcting that was not enough: a ligand-only classifier still separates actives from the matched decoys almost perfectly (Section 2.9.1), because the analogue exclusion rule places the two classes in disjoint regions of fingerprint space. Any evaluation of a score correction method is only as trustworthy as the comparison set that it is measured against, and property matching alone does not make that set difficult. We recommend reporting the active-versus-decoy standardised mean differences and, as a routine sanity check, the performance of a structure-free classifier on the same task. Absolute enrichment on a constructed benchmark should not be equated with protein-specific recognition.

**Pose correctness and score usefulness are different issues.** Cognate ligand redocking reproduced the crystallographic mode as the top-ranked pose at one of five targets (Section 2.9.2). This is a plausible mechanistic contributor to the absence of a potency signal (a score computed on the wrong binding mode has no particular reason to track affinity), but it is a contributor, not the explanation; we have not shown that the docked pose of every ChEMBL active is wrong, and SERT, where the pose is demonstrably right, still shows no usable ranking. Two further distinctions matter for interpretation. Reproducibility is not correctness: the NMDA pore solution was highly reproducible across seeds and consistently displaced from the crystallographic mode, so a seed-stability check must never be reported as pose validation. Moreover, pose generation and pose selection fail independently: for σ_1_, the correct pose was always sampled and merely mis-ranked, and rescoring the full pose list rather than the top-ranked one repaired it without additional docking, whereas for three other targets, the correct pose was never generated, and no rescoring could recover it.

**Assay–site matching is a precondition, not a refinement.** If the docked pocket is not the site that the reference assay measures, a score-versus-experiment comparison is uninterpretable whatever the scoring function. The NMDA case study (Section 2.5) shows the failure mode concretely, and the systematic classification (Section 2.9) shows that it is not hypothetical: one of our five comparison sets clearly refers to a different readout from the pocket that we docked. Site matching proved not to be the reason why ranking fails (restricting to site-matched compounds with rebuilt decoys moved ρ by less than 0.04), but that is a conclusion that we could only reach because a control analysis was run.

**Regarding what the negative probe does and does not license,** TFE-Rhy is an experimentally characterised negative probe that is held out from all fitting, normalisation, threshold setting, and benchmark construction procedures; its behaviour—consistently lowered but rarely formally flagged—indicates that property-matched correction is best used to shift such compounds toward the inactive tail and re-rank them, not as a hard classifier. The strict fifth-percentile criterion “caught” the probe at only one target and may be too cautious for large, flexible molecules; a looser operating point or a second independent filter such as the CNN pose score would “catch” more compounds at the cost of demoting genuine actives. We tested the second option directly (Section 2.8) and found it to be target-specific rather than general, so the appropriate threshold depends on the downstream economics of a wasted synthesis against a missed lead. The pooled size analysis (Section 2.4) sharpens the failure mode: it is *not* the over-penalisation of large molecules, since large actives retain a positive RDAS, but a loss of active/inactive discrimination **within** the large-molecule range, which is precisely where natural-product-like leads are sought. One compound assayed once in duplicate at five sites is a case study and not evidence that the score fails systematically across a chemotype.

**This study does have limitations.** Five constraints established by the control analyses in Section 2.9 bound how these results may be read. The benchmark is largely solvable without structure, so absolute enrichment is not evidence of protein-specific recognition. The structural hypothesis is unreliable for most targets, so pose failure is a plausible mechanistic contributor to the absence of ranking. The GABA_A_ active set is dominated by one data source and is reported with stratification rather than as the strongest evidence of anti-correlation. Per-compound scores are not exactly reproducible, because the seed of Uni-Dock was not fixed in the production run, although the aggregates are stable. Moreover, NMDA is weak on several independent axes at once—scaffold redundancy, low ChEMBL target-assignment confidence, three-layer species inconsistency, and failed pose recovery—so conclusions resting principally on it are the least grounded.

Further limitations are as follows: We only considered five targets, predominantly CNS binding sites. We employed one docking-engine family and one CNN rescorer—the default ensemble released with GNINA v1.3.2—without substitution or retraining, so nothing here speaks to convolutional scoring functions as a class. We adopted a specific null-construction protocol. Residual differences in NP-likeness and related unmatched descriptors remained between actives and decoys, and building decoys matched to such descriptors from a general drug-like library is difficult, so a small residual physicochemical confound cannot be excluded. Ranking was evaluated only among pChEMBL ≥ 7 actives: range restriction may attenuate observable rank correlation and limits generalisation beyond this high-potency range, and we cannot infer performance across the excluded pChEMBL 5–7 interval. Macrocyclic ligands were treated as rigid and receptors as rigid after heavy-atom reconstruction, choices which are the most consequential for large, conformationally complex ligands. CNN_VS was evaluated post hoc on poses generated and ranked by CNNaffinity. Generalisation beyond these targets, this scorer, and this chemical range is untested. We did not evaluate cofolding methods (for example Boltz, ESMFold or AlphaFold 3). These predict a complex for a given ligand rather than providing a calibrated score for ranking a library of the size benchmarked here, and in AlphaFold 3’s case, the terms of use restrict such an application; a cofolding comparison is therefore a study of pose prediction rather than a substitute for the present benchmark, and it is the test our pose results most directly motivate [12].

**Recommendation: A tiered workflow.** For the chemical and target range studied here, we recommend the following: (i) docking scores should be used for shortlist triage only after enrichment has been validated under a library composition relevant to the intended application (as neither the present benchmark nor the correction establishes such performance in realistic large libraries), with property-based de-biasing being treated as a target-specific optional layer whose benefit is demonstrated rather than assumed; (ii) **assay–site matching should be a mandatory precondition** for any score-versus-experiment comparison; (iii) potency prioritisation should be carried out by physics-based free-energy methods (FEP) or wet-lab dose–response, **not** raw docking score; and (iv) same-series (SAR) ranking should only be performed *after* experimental anchoring. These recommendations convert the present negative result into an operating procedure: docking is a triage filter, not a potency predictor, and must be followed by an experimental or physics-based anchor before any ranking is trusted. This is consistent with fixed-budget comparisons in which docking contributed complementary structural information but recovered fewer actives than ligand-based prioritisation when both were allowed the same number of compounds [2], and it argues for deploying docking where its contribution is orthogonal rather than where it is expected to be quantitative. Property-matched null correction is a low-cost optional adjustment whose utility should be demonstrated for the intended benchmark or screening context, rather than assumed; it is not a route to quantitative affinity.

## 4. Materials and Methods

### 4.1. Targets, Structures, and Assay–Site Matching

Five CNS targets were selected because each has (a) a well-characterised holo structure, (b) abundant binding data in ChEMBL, and (c) an assay-matched experimental measurement available for the experimentally characterised negative probe. For each target, the docking site was selected to correspond to the binding site probed by the radioligand in the associated binding assay, because a benchmark where a convenient pocket is docked and compared with an assay performed at a different site results in a meaningless correlation (Section 2.5).
TargetPDBCo-Crystal Ligand/SiteAssay-Matched RadioligandSERT [13]5I6Xparoxetine, orthosteric transporter site[^3^H]paroxetineGABA_A_ [14] (α_1_β_2_γ_2_)6X3Xdiazepam, benzodiazepine site[^3^H]Ro-15-1788 (flumazenil)5-HT_1A_ [15]7E2Zaripiprazole, orthosteric pocket[^3^H]8-OH-DPATσ_1_ (SIGMAR1) [16]5HK1PD144418, σ_1_ site[^3^H]pentazocineNMDA (pore) [17]5UN1MK-801 in the transmembrane channel pore[^3^H]MK-801NMDA (allosteric; site control only) [18]3QELifenprodil, GluN1/GluN2B ATD interface— (not assayed)

For 5-HT_1A_, an aripiprazole-occupied orthosteric structure (7E2Z) [15] was used as the box template in preference to a small endogenous-ligand pocket because it defines the full orthosteric volume; 7E2X and 7E2Y were inspected as alternates and not used, as their orthosteric volumes are defined by smaller ligands. Docking-box centres and dimensions for all targets are given in Supplementary Table S1.

SERT contains a central orthosteric (S1) binding site and a secondary extracellular allosteric (S2) site. Only S1 was considered here: the structural template 5I6X is paroxetine-bound, and the corresponding experimental comparator uses [^3^H]paroxetine, so S1 is the assay-matched site for this benchmark. S2 was, therefore, outside the assay-matched scope and was not evaluated; the site-matched sensitivity analysis in Section 2.9 is likewise restricted to S1-relevant records.

Species provenance was checked at three levels: the structural template, the ChEMBL comparison set, and the assay of the negative probe TFE-Rhy (2,2,2-trifluoroethyl rhynchophylline; Section 4.5) (Supplementary Table S2d). SERT, GABA_A_, 5-HT_1A_, and σ_1_ are human at all three levels. Regarding NMDA, the [^3^H]MK-801 pore site is conventionally assayed in rodent brain membranes: the TFE-Rhy assay used Wistar rat brain, and the ChEMBL pore-site actives are correspondingly predominantly rat (67.8% of pore-site activity records annotated to *Rattus norvegicus* and 2.3% to *Homo sapiens*). The structural template 5UN1 is a *Xenopus laevis* GluN1/GluN2B construct, the standard crystallographic form for this receptor. The assay and the comparison set against which the false-positive probe is ranked are, therefore, species-consistent, while the structural template is not; the channel-pore blocker site is conserved across these species.

### 4.2. Ligand Sets and Property-Matched Nulls

**Selection:** For each target, ChEMBL [19] binding activities were retrieved by target identifier (SERT: CHEMBL228; GABA_A_ α1: CHEMBL1962; 5-HT_1A_: CHEMBL214; σ_1_: CHEMBL287; NMDA as described below), restricted to *standard_type* in {Ki, Kd, IC_50_} and to binding assays (*assay_type* = “B”) carrying a pChEMBL value, and aggregated to one median pChEMBL value per compound. Compounds were then classified as **active** when pChEMBL ≥ 7 and **inactive** when pChEMBL ≤ 5; the intermediate band was excluded from both sets. Where more than 800 actives remained, the set was subsampled to 800 before docking, without replacement, *using Python 3.12.13’s random.sample* seeded with *random.seed(42)*; the exact procedure and the script used to perform it have been deposited. With this method, we compare confidently active against confidently inactive compounds at the cost of truncating the activity range available to the ranking analysis (Section 3, Limitations). Table 6 shows the counts at each stage.

**Curation checks:** No explicit *standard_relation* filter was applied, which is without consequence. ChEMBL assigns a pChEMBL value only to exact-relation measurements, and every record entering our sets carried “=” (Table S12). Re-deriving potencies under stricter curation—i.e., adding the relation filter and excluding records with *data_validity_comment* or flagged *potential_duplicate*—changed the within-active ρ by at most 0.007 and removed two compounds in total (Table S13). The assay–target confidence score of ChEMBL was 8 or 9 for every contributing record for SERT, GABA_A_, 5-HT_1A_, and σ_1_, but the median score was 4 for NMDA, whose pore set comes from channel-blocker assays on rodent brain homogenate (Table S22). We did not filter by confidence, since that would have removed the one target whose assay site we could match exactly, but the low value for NMDA is reported along with its other weaknesses in Section 3. Data are from **ChEMBL 37** (released 1 May 2026), retrieved 11–12 June 2026; the full query parameters and retrieved assay identifiers have been deposited.

An important limitation follows from this procedure. For NMDA, the retrieved set was re-filtered by pore-site record ([^3^H]MK-801/PCP/TCP; 256 assays), so the comparison set matches the docked site. **For the other four targets, no assay-level site filters were applied**, and the retrieved records are those that, in ChEMBL, are associated with the target identifier rather than with the specific pocket that was docked. Therefore, we classified the assay description of every activity record underlying our active sets (Table S3). Each record is assigned to exactly one of four mutually exclusive classes according to the radioligand or probe that it names: **docked pocket**, when it refers to a docked pocket; **other site**, when it refers to another structurally distinct site of the same protein; **functional**, when it refers to a site-agnostic functional readout; and **unclassified**, where no diagnostic probe is named. The denominator is the set of records that entered the active sets, that is, those from ChEMBL binding assays (*assay_type* = “B”) for the docked actives of each target.

Site correspondence is good for NMDA (92.9% of records report on the docked pocket), 5-HT_1A_ (72.9%), SERT (56.7%), and σ_1_ (52.9%, with a further 16.3% naming a σ_2_ or non-selective probe); the unclassified remainder at those targets consists of descriptions that name no diagnostic probe rather than records identifiable with a different site, and all their records are annotated to human targets.

GABA_A_ is the exception and by a wide margin. Only **5.0%** of its records report on the benzodiazepine pocket ([^3^H]flunitrazepam, [^3^H]Ro 15-1788, [^3^H]zolpidem, and diazepam), while **84.8%** describe electrophysiological measurements at α1-containing receptors rather than radioligand binding. The GABA_A_ comparison set is, therefore, heterogeneous in both assay format and site, even though the docking box, the co-crystal template (6X3X, diazepam) and the TFE-Rhy assay ([^3^H]Ro 15-1788) all correspond to the benzodiazepine site. Because this is exactly the kind of benchmark-design confound that the present study is about, we tested directly whether it accounts for the ranking result of GABA_A_ rather than assuming that it does. Using the existing docking scores, the within-active correlation was recomputed on assay-defined subsets of the GABA_A_ actives (Section 2.1, Table S4); no redocking was required, since the analysis restricts the compounds entering the correlation rather than changing how they are scored.

Activity values are ChEMBL pChEMBL medians derived from heterogeneous binding measurements (Ki, IC_50_, and Kd; per-target composition in Supplementary Table S5). Therefore, we use “experimental potency” rather than pK_i_ for the pooled sets. No IC_50_→Ki conversion was applied, as the assay-specific radioligand concentration and Kd values required for Cheng–Prusoff correction [20] were not uniformly available; a Ki-only sensitivity analysis is reported in Section 4.8 and Table S5. Property-matched **nulls** (decoys) were drawn from a 30,000-molecule drug-like ChEMBL background pool (MW, 180–700; non-null AlogP, assembled with *random.seed(7)*). The pool was accumulated at random query offsets, a procedure that cannot be replayed against a different ChEMBL release; reproducibility is, therefore, secured by depositing the pool actually used, with its SHA-256 checksum. The decoy sets comprise 5230 unique molecules representing 6241 target-specific decoy assignments, and each was drawn from this pool. We initially selected nulls based on binned molecular weight (150 per 50 Da bin). An audit of this set (Section 2.2; Figure 4) showed that although matched in MW, the decoys differed markedly from the actives in polarity and flexibility (standardised mean difference (SMD), the difference in group means divided by the pooled standard deviation; |SMD| ≈ 0.8–1.2 in TPSA, H-bond acceptors, and rotatable bonds). Therefore, we **redesigned** the null sets, matching each active to its nearest background-pool molecules in a seven-descriptor space (molecular weight, cLogP, TPSA, H-bond donors, H-bond acceptors, rotatable bonds, and formal charge), each descriptor standardised to the pool mean and standard deviation. The nearest neighbours were identified based on Euclidean distance in this standardised space using a k-d tree, requesting k = round(N/n_actives_) decoys per active, where N was set to the size of the corresponding first-generation null set so that the two decoy generations are comparable in size; selection proceeded greedily without replacement, skipping excluded molecules and molecules already assigned to another active. Topological analogues of the actives were excluded (Morgan fingerprints, radius 2, 1024 bits; Tanimoto ≥ 0.35 to any active in the target’s set) [8,21]. These matched decoys were docked and scored under an identical protocol. Both decoy generations are reported (Section 2.2). After redesign, the seven matched descriptors had |SMD| ≤ 0.44; residual differences remained only in fraction-sp^3^ carbon, aromatic-ring count, and NP-likeness—descriptors not used for matching on which residual differences across the active sets thus remained. The final docked counts per target (active/matched decoys) were as follows: SERT: 793/1493; NMDA(pore): 194/950; GABA_A_: 273/855; 5-HT_1A_: 799/1563; σ_1_: 792/1380 (6241 matched decoys in total). The corresponding ChEMBL inactives were 124/210/2/96/62, respectively. The first-generation MW-binned null sets comprised 1499/1377/1050/1500/1349 molecules (6775 total). The negative probe (TFE-Rhy) was **never** included in any null set. It was excluded from all steps (normalisation, model fitting, decoy construction, and threshold setting), so no quantity reported for it was informed by its own score.

### 4.3. Docking and Scoring

Ligands were protonated at pH 7.4 (Dimorphite-DL [22]), embedded in 3D with RDKit 2025.09.6 (ETKDGv3) [23] and MMFF-minimised, and converted into PDBQT with Meeko [24] (macrocycles treated as rigid). Receptors were rebuilt at the heavy-atom level with PDBFixer [25] and converted into rigid PDBQT with Meeko (SERT, GABA_A_, and 5-HT_1A_) or OpenBabel [26] (NMDA and σ_1_). The preparation was as follows, and the scripts used to perform it have been deposited unmodified.

*For chain and heteroatom selection*, only the first model of each entry was used. Protein chains were retained if at least one atom lay within 14 Å of the box centre; all other chains were discarded. **All HETATM records were removed** (crystallographic waters, ions, co-crystallised ligands, lipids, detergents, and glycans alike), so docking was performed against an apo, water-free, and cofactor-free receptor. None of the five sites has a catalytic metal or an obligate cofactor in the pocket. Where alternate locations were present, altloc A (or blank) was kept.

*For rebuilding and protonation,* PDBFixer replaced non-standard residues and rebuilt heavy atoms missing from residues that were present. **Whole missing loops were deliberately not modelled** (*missingResidues* was set as empty), since modelled loops would add unvalidated structure near the pocket. Receptors were written as heavy-atom only; **hydrogens and receptor protonation states were assigned by Meeko at typing time under its default rules**, and no manual adjustment of side-chain protonation, histidine tautomers, or titratable residues was performed. Ligand protonation was handled separately at pH 7.4 (Section 4.2).

*For box definition*, each box was centred on the centroid of the heavy atoms of the co-crystallised ligand that defines the site (the first chain/residue instance), with 8 Å of padding beyond that ligand’s bounding box on each side, and each side length was clamped to between 18 and 30 Å. The resulting centres and dimensions are shown in Table S1.

*For sampling*, Uni-Dock v1.1.0 was run in *--search_mode balance*, which selects that version’s built-in exhaustiveness and search-step settings; we did not override them, so the preset is the reproducible specification. The docking of the benchmark compounds used *--num_modes 1*, so a single top-ranked pose per ligand was carried forward to CNN rescoring. In the redocking and re-ranking experiments in Section 2.9.2, we used --*num_modes 9*; because *--num_modes* controls how many poses are written rather than how the search proceeds, the rank-1 pose from those runs is the pose that the benchmark pipeline would itself have used. Poses were generated with Uni-Dock (balance mode, GPU) [27] and rescored with GNINA v1.3.2 (*--cnn_scoring rescore*; CNNaffinity taken as the score) [11,28,29]. Rescoring used the default CNN ensemble that ships with the v1.3.2 binary (*--cnn* left at *default*); no models were substituted, retrained, nor fine-tuned, and no ensemble members were selected or weighted by us. Our conclusions, therefore, apply to this specific released model as used out of the box, not to convolutional scoring functions as a class, and a differently trained network could behave differently. The **random seed of Uni-Dock was not fixed in the production run**, so per-compound scores are not exactly reproducible; the magnitude of this variation and its effect on the aggregate conclusions are quantified in Section 2.10. In all analyses added during revision, *--seed* was set explicitly. AutoDock Vina scores (via Uni-Dock v1.1.0) [30,31] were retained as a sensitivity check (Table S6). Compounds for which CNN rescoring failed were excluded from all analyses; on the production A100 hardware, no rescoring failures occurred (Section 2.6), so this exclusion removed no compounds from the reported results. A total of 10,130 receptor–ligand pairs were docked in the initial run (actives, inactives, MW-binned decoys, and probes), and a further 6241 seven-descriptor-matched decoys were docked in the redesigned run under an identical protocol; the CNN-rescoring failure rate was 0 on the production A100 hardware (Section 2.6).

### 4.4. The Reference-Distribution-Adjusted Score (RDAS) and Its Circularity-Free Evaluation

For each compound *i*, the reference-distribution-adjusted score (RDAS) is the z-score of its CNNaffinity relative to the null distribution within its molecular-weight bin:

RDAS_i_ = (CNNaff_i_ − μ(null_ref, bin(i)))/σ(null_ref, bin(i))

Bins are defined as ⌊MW/50⌋, i.e., anchored at 0 Da with edges at 50 Da intervals, not at the minimum observed molecular weight. The global fallback is the z-score against the entire *null_ref* set, applied when a compound’s bin contains fewer than 10 reference molecules or has zero variance. To avoid circularity between the correction and its evaluation, the null set was split 50/50 into a **normalisation half** (*null_ref*, used to compute μ and σ) and a **disjoint held-out half** (*null_eval*, used only for AUC). Evaluation decoys, therefore, never enter the z-score.

Because a single partition is itself a source of variance, all enrichment results reported here are summarised over 200 independent random 50/50 partitions, which were drawn by permuting the decoy index with a NumPy *Generator* seeded as *numpy.random.default_rng(7)* and splitting at the midpoint; they are given as the mean with the 2.5–97.5 percentile interval across partitions. We additionally report the proportion of partitions in which the correction improved the AUC. Point estimates from any single partition are not reported, as the between-partition spread is comparable to the effect being estimated in terms of magnitude.

### 4.5. The Experimentally Characterised Negative Probe

**Figure 10:**
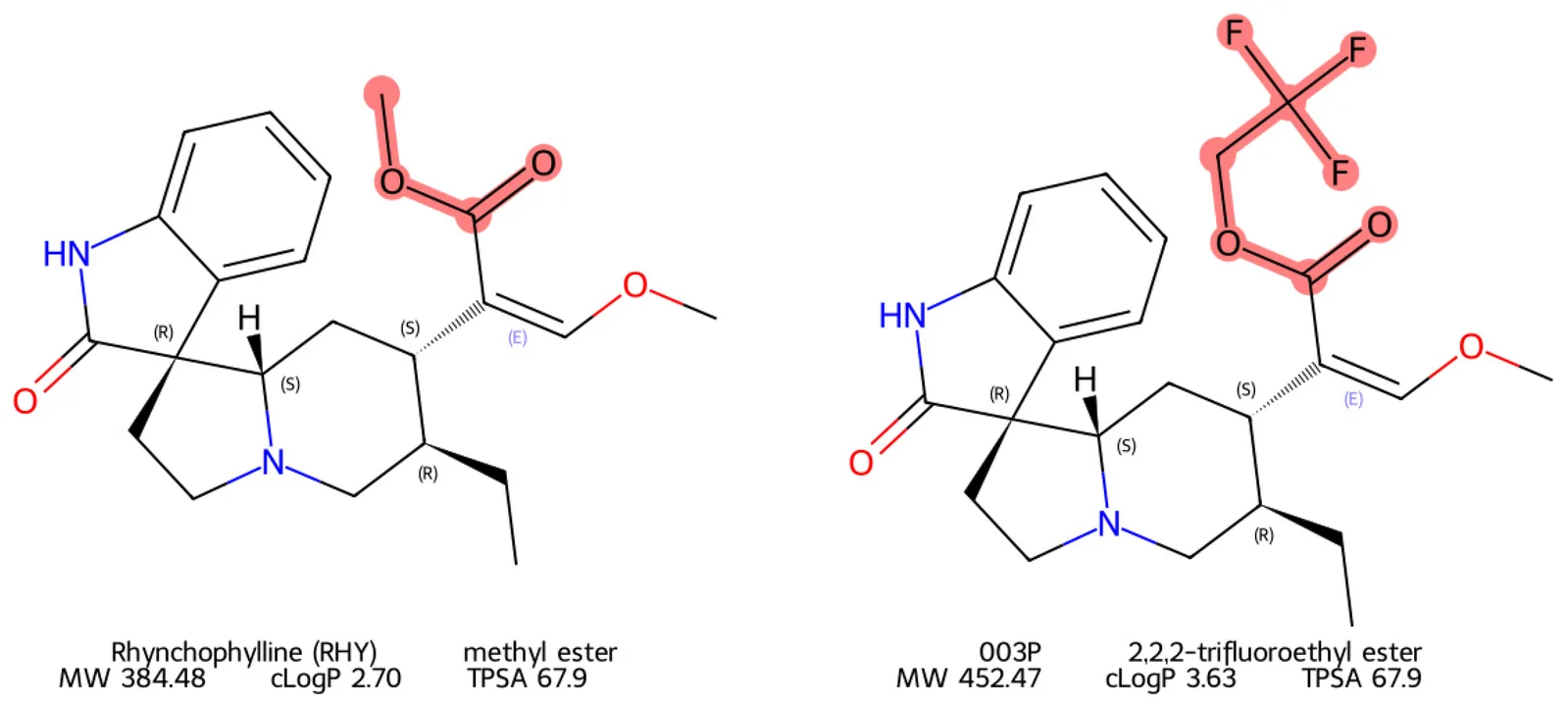
Structures of the parent alkaloid rhynchophylline (RHY) and the negative probe TFE-Rhy. The two differ only in the ester alkyl group (highlighted): methyl in RHY and 2,2,2-trifluoroethyl in TFE-Rhy. The substitution increases the molecular weight (384.48 → 452.47) and the calculated lipophilicity (cLogP 2.70 → 3.63) while leaving the polar surface area unchanged (TPSA: 67.9 Å^2^), that is, it moves the molecule along the axis on which property-dependent score bias is expected to be the strongest.

TFE-Rhy (2,2,2-trifluoroethyl rhynchophylline; internal laboratory code: 003P, the identifier appearing in the deposited assay report in Supplementary File S1) is the 2,2,2-trifluoroethyl ester of rhynchophylline, the principal alkaloid of *Uncaria rhynchophylla*. The parent scaffold has documented CNS pharmacology—non-competitive NMDA antagonism without interaction at the polyamine, Zn^2+^, proton, or redox sites [10,32]—which motivated the target panel used here. The two compounds differ only in the ester alkyl group (Figure 10), a substitution that raises size and lipophilicity while leaving polar surface area unchanged; it moves the molecule along the axis on which property-dependent score bias is expected to be the strongest. Both were scored.

TFE-Rhy was profiled at all five target sites in the radioligand binding assays (10 µM, duplicate; Eurofins Panlabs Discovery Services Taiwan, Ltd., New Taipei City, Taiwan). The percent inhibition of specific binding was −7% (GABA_A_ benzodiazepine site), 4% (NMDA MK-801 site), 4% (5-HT_1A_), 15% (σ_1_), and −2% (SERT); the laboratory’s significance criterion was ≥50%, and no assays met it. The performance of concurrent reference compounds was within approximately two-fold of historical values in every batch. Conditions, radioligand identities, and concentrations are shown in Supplementary Table S2, and the laboratory report is in Supplementary File S1.

**The probe was never used to tune anything.** TFE-Rhy was excluded from every null set, from all normalisation and model fitting, from decoy construction, and from threshold setting; its label entered the analysis only when its percentile was reported. **It is not a prospective prediction**: the assay report predates the docking, so we describe it as an experimentally characterised negative probe rather than a forecast. We also describe it as binding-negative *under the tested conditions*, not as pharmacologically inactive; single duplicate determination at one concentration does not exclude activity at untested sites, concentrations, or functional endpoints. The chronology and the reason why a measured negative is a different and rarely reported kind of control from the hit-rate studies that dominate prospective docking validation are given in Supplementary Note S1.

### 4.6. Metrics

- **Ranking (primary):** Spearman ρ between score and experimental potency, computed over actives only. Δρ = ρ(RDAS) − ρ(raw).
- **Enrichment:** ROC AUC, actives versus held-out property-matched nulls (*null_eval*), and actives versus ChEMBL inactives where n(inactive) ≥ 50. As in virtual screening-oriented summaries, we additionally report enrichment factors at the top 1/5/10% (EF1%, EF5%, and EF10%, respectively), BEDROC (α = 20) [33], and PR-AUC on actives versus held-out matched nulls (Section 2.2; Supplementary Table S7).
- **False-positive probe:** TFE-Rhy (and RHY) percentile *within actives*, raw versus RDAS scoring; “caught”, and below the fifth percentile of actives.
- **Size control:** Mean RDAS stratified by size (large/small MW) × activity (active/inactive).

### 4.7. Uncertainty and Robustness

Rank correlations are reported with permutation *p*-values (5000 random shuffles of the activity labels) and bootstrap 95% confidence intervals (2000 resamples of actives). Permutation *p*-values are two-sided, computed as (1 + #{|ρ_perm_| ≥ |ρ_obs_|})/(1 + n_perm_) with n_perm_ = 5000; the smallest attainable value is, therefore, 2 × 10^−4^. Bootstrap intervals are 2.5–97.5 percentile intervals. Random seeds are fixed and given in the deposited code. Enrichment AUCs and their raw-versus-RDAS differences (ΔAUC) were assessed for robustness with bootstrap resampling (2000 paired resamples of actives and nulls) and, to confirm that the gain was not an artefact of a single null partition, with the 50/50 normalisation/evaluation split (Section 4.4) repeated 200 times with independent random partitions; in Table 2, we report ΔAUC summarised over 200 partitions. As a sensitivity check on the *form* of the correction, the MW-binned z-score was compared against a continuous MW-regression residual (CNNaffinity de-trended on a linear fit of CNNaffinity to molecular weight estimated on the null set). Decoy property matching was audited by the standardised mean difference (SMD = (mean_active − mean_decoy)/pooled SD) on ten descriptors; the MW-binned and seven-descriptor-matched decoy sets are compared in Figure 4.

### 4.8. Additional Sensitivity Analyses

Three analyses were added to test whether the ranking result is an artifact of experimental data quality and whether a richer null model behaves differently from the MW-binned z-score; five further analyses, described at the end of this section, were conducted to test whether the benchmark itself, the chemical redundancy of the active sets, the assay–site correspondence, the correctness of the docked poses, or the stochasticity of the docking search can account for the results.

*For data-quality sensitivity (ranking),* to separate model behaviour from experimental-data quality, the within-active Spearman ρ was recomputed on three nested subsets of the actives: single-assay-type actives (ChEMBL standard type recorded as “Ki” only; compounds with mixed-type records such as “IC50|Ki” were excluded from this subset rather than counted as Ki), replicated actives (n_meas ≥ 2), and their intersection. The number of actives in each subset is reported (Supplementary Table S5); subsets below ≈20 compounds are flagged as unstable and not interpreted.

*For multivariate null correction*, two additional null models were fit on the normalisation half of the matched decoys only (no active labels used) and applied to the actives and to the held-out evaluation decoys: (i) ordinary least squares of CNNaffinity on molecular weight, cLogP, and TPSA, taking the residual as the corrected score; (ii) a random forest regressor (100 trees; maximum depth, 6) of CNNaffinity on six descriptors (MW, cLogP, TPSA, HBD, HBA, and rotatable bonds), residual as the corrected score. Descriptors for actives and decoys were computed from SMILES with RDKit 2025.09.6 using MolWt, Crippen MolLogP, TPSA, CalcNumHBD, CalcNumHBA, and CalcNumRotatableBonds; both models were fitted with scikit-learn 1.9.0 [34] on the normalisation half of the decoys only, with no active labels used at any stage. Descriptor bias was summarised by the standardised coefficients of a linear fit of CNNaffinity to the six descriptors over pooled decoys (Supplementary Table S8). All splits used a fixed random seed, stated in the deposited code.

*Ligand-only control.* To determine how much of the active-versus-decoy separation requires structural information at all, a classifier was trained on the same compounds using only two-dimensional ligand features: twelve physicochemical descriptors, ECFP4 fingerprints (Morgan, radius 2, 1024 bits), and the two combined, with logistic regression and random forest. Performance was estimated by using stratified five-fold cross-validation **grouped on a Bemis–Murcko scaffold**, so that no scaffolds appeared in both a training fold and a test fold. As a diagnostic, the Tanimoto similarity of each decoy to its nearest active and that of each active to its nearest other active were computed on the same fingerprints.

*Scaffold redundancy and scaffold-aware resampling.* Bemis–Murcko scaffolds (standard and generic) were enumerated for every active set, and the composition was summarised by the number of distinct scaffolds, the share of molecules in the largest families, and the effective number of scaffolds exp(H) from the Shannon entropy of the family-size distribution. All interval estimates were then recomputed with the **scaffold family, not the molecule, as the resampling unit** (cluster bootstrap, 500 replicates, each drawing its own held-out decoy partition), alongside the conventional molecule-level bootstrap.

*Site-matched benchmark.* Every ChEMBL assay behind every active was classified according to the radioligand or probe named in its description referring to the docked pocket, a different site of the same protein, or a site-agnostic functional readout. Because the decoys had been matched to the full active set, **decoys were rebuilt for each site-matched subset** by re-running the matching procedure against the background pool, and the 1506 newly selected molecules were docked under an identical protocol; decoys already docked were reused. Enrichment was then recomputed under the held-out protocol. Where a subset changed the decoy–active ratio, early-recognition metrics were computed after downsampling the evaluation decoys to the original ratio, averaged over 200 draws.

*Cognate ligand redocking.* For each receptor, the co-crystallised ligand was rebuilt from SMILES—discarding its crystallographic coordinates—and redocked with an identical preparation, docking, and rescoring protocol. The symmetry-corrected heavy-atom RMSD from the crystallographic pose was computed in the receptor frame without superposition. Because the search is stochastic, each ligand was redocked under **50 explicit seeds**, and the distribution was reported; the number of seeds was chosen from the observed convergence of the estimates rather than by convention. Binding-site recovery was quantified as the fraction of residues within 4 Å of the crystallographic ligand that were also within 4 Å of the redocked pose. In a follow-up experiment, the complete nine-pose Uni-Dock output was rescored with GNINA, instead of only the Vina rank-1 pose, to separate pose generation from pose selection.

*Docking stochasticity.* A representative subset of each target (120 actives and 240 decoys) was redocked under five explicit seeds, and the aggregate quantities that support the conclusions—raw enrichment AUC and within-active Spearman ρ—were compared across seeds and against the archived production scores **for the same sampled compounds**.

## 5. Conclusions

Improving the calibration of a docking score is not the same as improving every decision taken based on it. In this benchmark, property-aware correction produced small, target-dependent shifts in decoy-benchmark enrichment and early recognition which were stable across normalisation splits but not robust to compound or scaffold resampling, and it did not recover within-active potency ranking at any target, because the underlying score carried little recoverable affinity information across the activity range tested. Because ranking was evaluated only among pChEMBL ≥ 7 actives, range restriction may attenuate observable rank correlation and limits generalisation beyond this high-potency range; we cannot infer performance across the excluded pChEMBL 5–7 interval. Apparent enrichment on such a benchmark is strongly benchmark-dependent and should not be equated with protein-specific recognition: a ligand-only classifier separated the same actives from the same decoys at ROC-AUC values of 0.959–0.993 using no structural information at all. Restriction to the highest-quality experimental data, multivariate null models, or GNINA’s virtual-screening composite did not change this. Three design lessons generalise beyond the present systems. Apparent gains from score correction are strongly dependent on how the comparison set is constructed, and property matching alone does not guarantee a challenging benchmark when active analogues are internally clustered but decoys are explicitly excluded from the same topological neighbourhood. Matching the docked pocket to the experimental radioligand site is a precondition for any score-versus-experiment comparison, though in this benchmark, it proved not to be the explanation for the ranking failure. Moreover, a score is only as meaningful as the structural hypothesis on which it is computed: cognate ligand redocking reproduced the crystallographic mode as the top-ranked pose at one of the five targets, so pose generation and pose selection represent a plausible mechanistic contributor to the absence of a potency signal. These results support separating shortlist enrichment from potency prioritisation but do not establish protein-specific screening performance in realistic large libraries. Docking scores used for triage should, therefore, be validated under library compositions appropriate to the intended decision and should not be treated as quantitative potency rankings; potency prioritisation belongs with dose–response measurement, physics-based free-energy methods, or experimentally anchored SAR. A recent comparison of physics-based, deep-learning-assisted and cofolding tools on coronavirus targets reports the same coupling from the other direction (the systems with the poorest pose prediction also gave the lowest enrichment factors, ROC-AUC and BEDROC [12]) and finds cofolding most accurate for pose recovery, which makes cofolding the natural next test of whether better complexes yield a more decision-relevant score.

## Figures and Tables

**Table 1:** Property bias strategies: what each corrects and evaluates.

Approach	Which Bias It Corrects	Circularity Control Analysis	Ranking vs. Experiment Tested	Enrichment Tested	Experimentally Characterised Negative Probe
Property-matched decoys (DUD-E-style) [3]	none (decoy construction only)	partial (depends on match quality)	no	yes	no
Ligand efficiency normalisation [4]	size (per heavy atom)	n/a (rule of thumb)	seldom	seldom	no
MW/property regression de-trending (Δ-ML-style) [5]	fitted property offset	rarely stated	rarely	yes	rarely
Property-matched null z-score, evaluated as shown in this work	property-dependent score offset via property-matched null z-score	yes (normalisation/evaluation null split)	yes (primary)	yes	yes (TFE-Rhy)

**Table 3:** Test B—TFE-Rhy percentile within actives (CNNaffinity; RDAS vs. matched decoys).

Target	TFE-Rhy Raw %ile	TFE-Rhy RDAS %ile	Below 5th %ile (Raw/RDAS)
SERT	20.2	8.4	no/no
NMDA (pore)	43.8	6.7	no/no
GABA_A_	41.4	12.8	no/no
5-HT_1A_	40.4	26.9	no/no
σ_1_	4.4	0.4	yes/yes

**Table 4:** CNN-rescoring failure rate by molecular weight band (A100, all targets).

MW Band	n	Failed	Fail Rate
<450	7070	0	0.0%
450–500	1112	0	0.0%
>500	1948	0	0.0%

**Table 5:** Robustness control analyses. Each aims to determine whether a specific feature of the study design, rather than the scoring function, could account for the reported results.

Control Analysis	Question	Key Result	Implication
Ligand-only classifier (Table S16)	Can the actives be separated from the decoys without any structural information?	ROC-AUC of 0.959–0.993 under scaffold-grouped CV, against 0.57–0.73 for docking on identical task	Absolute enrichment on this benchmark is not evidence of protein-specific recognition
Scaffold-aware resampling (Table S14)	Are the estimates inflated by families of close analogues?	Intervals widen to 1.06–2.77×; ρ conclusions are unchanged, and ΔAUC interval includes zero for all five targets	Chemical redundancy does not explain the ranking failure; it does remove the enrichment claim
Site-matched benchmark; decoys rebuilt and redocked (Table S17)	Does the comparison set report on the pocket that was docked?	ρ moves by less than 0.04 for every target	Assay–site mismatch is not the main reason why ranking fails
Strict curation (Table S13)	Is the result an artefact of activity data quality?	Two compounds removed in total; ρ changes by at most 0.007	Curation choices do not explain the result
Cognate ligand redocking; 50 seeds (Table S19)	Is the structural hypothesis on which the score is computed correct?	Crystallographic mode recovered as the top pose for one target of five	Pose failure is a plausible mechanistic contributor, not a confound
Docking stochasticity; 5 seeds (Table S21)	Are the aggregates stable under an unseeded search?	Per-molecule CNNaffinity spans 0.10–0.72 units; raw AUC varies with SD 0.007–0.022	Individual scores are not reproducible; the aggregates are

**Table 6:** Compound counts per target, from retrieval to the population that entered the benchmark. The excluded band is 5 < pChEMBL < 7. Losses between “after cap” and “scored” are ligand preparation or CNN-rescoring failures.

Target	Actives Retrieved	Excluded 5–7 Band	After Cap	Actives Scored	Inactives Scored
SERT	2940	2221	800	793	124
NMDA	898 †	858	194 †	194	210
GABA_A_	276	53	276	273	2
5-HT_1A_	3245	1870	800	799	96
σ_1_	1928	1208	800	792	62

† NMDA counts refer to before and after re-filtering by pore site (see below).

## Data Availability

All benchmark data and analysis code are openly available on the Open Science Framework (OSF) at https://osf.io/uvp84/ (accessed on 24 October 2026; DOI: 10.17605/OSF.IO/UVP84). The deposit contains, per target, ligand sets with SMILES, experimental potency values, activity labels, and computed physicochemical descriptors; both decoy generations (molecular-weight-binned and seven-descriptor property-matched sets), together with the ≈30,000-molecule background pool; per-compound docking and rescoring outputs (GNINA CNNaffinity, CNN pose score, AutoDock Vina score, and reconstructed RDAS); docking-box geometries and receptor preparation reports; and the experimentally characterised negative probe (TFE-Rhy) and its parent (rhynchophylline). All analysis scripts—decoy matching, the RDAS correction, the enrichment and ranking analyses, and the sensitivity analyses on data quality, early-recognition metrics, and multivariate nulls— are included. The deposit also holds the following controls added in revision: the ligand-only classifier and its nearest-neighbour diagnostic, the Bemis–Murcko scaffold audit and scaffold-aware resampling, the rebuilt site-matched decoys and their docking outputs, the cognate ligand redocking and pose re-ranking experiments, the docking stochasticity experiment, and the curation provenance and attrition audits. Source receptor structures are public and cited by PDB accession (5I6X, 6X3X, 7E2Z, 5HK1, 5UN1, and 3QEL) rather than re-deposited; the prepared receptors and the redocked poses are included. The deposit also contains a *repro*/directory. *ENVIRONMENT.md* records the full computational environment: the GPU and CPU stacks, all package versions, the CUDA-12 runtime that GNINA requires, and a table stating which steps are seeded and which are not. *COMMANDS.md* gives the exact command lines for every stage, *requirements.txt* pins the analysis packages, and *reproduce.sh* regenerates the tables and figures from the deposited data on CPU alone, without network access. A log of a complete successful run has also been deposited. Two guards protect the deposit against the error that most affected this work. *decoy_generation_guard.py* asserts the expected decoy counts, so any script that accidentally loads the superseded molecular-weight-binned pool fails immediately rather than silently reporting inflated enrichment; *verify_delta_auc.py* reproduces the ΔAUC values reported in Table 2 and prints, side by side, the values that the wrong decoy generation would give. Scripts that query ChEMBL retry on failure and raise rather than returning a truncated result.

## Supplementary Materials

The following supporting information can be downloaded at: https://www.mdpi.com/article/10.3390/ijms27219652/s1.
