# Supplementary material for: A Decision-Centric Benchmark of Enrichment and Potency Ranking in CNN-Rescored Molecular Docking
Source: Int J Mol Sci. 2026 Oct 28;27(21):9652. doi: 10.3390/ijms27219652 (PMC13762515; doi:10.3390/ijms27219652)
Supplement: Supplementary file 1 [file ijms-27-09652-s001.zip › ijms-4607028-supplementary.pdf]

Supplementary Materials

A Decision-Centric Benchmark of Enrichment and Potency Ranking in CNN-Rescored Molecular Docking

Yen-Hsun Lai and Hsiang Chi Huang

Manuscript ID: ijms-4607028

Supplementary information

Supplementary Table S1. Docking-box geometry. Box centres and dimensions (Å), with the co-crystal ligand used to define each box, are deposited with the analysis code and data (see Data and code availability).

Supplementary File S1. Redacted original laboratory report. The complete nine-page study report issued by Eurofins Panlabs Discovery Services Taiwan (study TW04-0020744, 1 June 2026) is supplied as a Supplementary File. The sponsor's postal address and the contact telephone number have been removed; no scientific content has been altered, and the removed text is not recoverable from the file. The transcription below is provided for convenience and for machine readability.

Supplementary Table S2. TFE-Rhy radioligand binding assay (Eurofins Panlabs Discovery Services Taiwan, study TW04-0020744).

(a) Results. Test article: rhynchophylline derivative (003P in the study report; denoted TFE-Rhy in this work), Panlabs # 1300652, code MED-1, batch ET111402-8, MW 452.47. All assays: 10 µM, duplicate, 1.0% DMSO vehicle, significance criterion ≥ 50% inhibition (not met at any site). Percent inhibition of specific binding is quoted as reported in the `% Inh.` column of the study report; the companion machine-readable data file gives the same values to one decimal place (−7.3, 4.3, 3.7, 14.6 and −2.5 respectively).

Cat #	Assay	Species	Source	Radioligand	% inhibition
226640	$GABA_A$ α1β2γ2, Ro 15-1788 site	Human	—	[^{3}H] Ro-15-1788	−7
233010	Glutamate, NMDA, MK-801 site	Rat	brain (minus cerebellum)	[^{3}H] MK-801	4
271110	Serotonin 5-HT_{1A}	Human	recombinant CHO-K1	[^{3}H] 8-OH-DPAT	4
299034	Sigma $σ_1$	Human	Jurkat cells	[^{3}H] pentazocine	15
274030	Serotonin transporter	Human	recombinant HEK-293	[^{3}H] paroxetine	−2

Cat #	Assay (SERT)	Species	Source	Radioligand	% inhibition

(b) Assay conditions.

Cat #	Radioligand conc.	Non-specific ligand	Incubation	Buffer	Kd †
226640	‡	‡	60 min @ 4 °C	‡	‡
233010	5.0 nM	10.0 μM dizocilpine	3 h @ 25 °C	5 mM Tris-HCl pH 7.4	12.0 nM
271110	1.50 nM	10.0 μM metergoline	60 min @ 25 °C	50 mM Tris-HCl pH 7.4, 0.1% ascorbic acid, 0.5 mM EDTA, 10 mM $MgSO_4$	2.0 nM
299034	15.0 nM	10.0 μM haloperidol	2 h @ 37 °C	50 mM Tris-HCl pH 8.0	16.0 nM
274030	0.4 nM	10.0 μM imipramine	60 min @ 25 °C	50 mM Tris-HCl pH 7.4, 120 mM NaCl, 5 mM KCl	0.078 nM

† Historical values as reported by the testing laboratory. ‡ Not specified in the materials received; the assay is a catalogue-defined commercial service (Cat # 226640) and its concurrent reference compound performed to specification (panel c).

(c) Concurrent reference compounds.

Cat #	Reference compound	Historical IC50	Concurrent IC50
226640	Diazepam	0.073 μM	0.073 μM
233010	Dizocilpine ((+)-MK-801)	7.10 nM	5.29 nM
271110	Metergoline	4.10 nM	4.21 nM
299034	Haloperidol	2.0 nM	3.26 nM
274030	Fluoxetine	8.60 nM	15.6 nM

(d) Species provenance across the three layers.

Target	PDB	Structure organism	ChEMBL comparison set	TFE-Rhy assay

Target	PDB	Structure organism	ChEMBL comparison set	TFE-Rhy assay
SERT	5I6X	*Homo sapiens*	human (CHEMBL228)	human (HEK-293)
$GABA_A$	6X3X	*Homo sapiens*	human (CHEMBL1962)	human
$5\text{-}HT_{1A}$	7E2Z	*Homo sapiens*	human (CHEMBL214)	human (CHO-K1)
σ_1	5HK1	*Homo sapiens*	human (CHEMBL287)	human (Jurkat)
NMDA (pore)	5UN1	*Xenopus laevis*	*Rattus norvegicus* 67.8%, *Homo sapiens* 3.3%	*Rattus norvegicus*

Supplementary Note S1. Chronology of the negative probe, and what "held-out" means here. The binding assays were commissioned and completed before the docking benchmark was assembled: the test article was received on 13 May 2026, the assay report was issued on 1 June 2026, and receptor preparation, docking and rescoring were performed on 11–12 June 2026. The computational predictions were therefore not frozen in advance of an unknown experimental outcome, and we do not describe this as prospective validation. What the design provides instead is a compound carrying a measured, externally determined binding-negative label that was never used to select targets, construct decoys, fit the correction or set any threshold.

This is a different and less commonly reported control from the prospective studies that dominate the docking literature, which are generally reported as hit rates among top-ranked compounds. At the D4 dopamine receptor, for example, 549 compounds sampled across high-, mid- and low-ranking docking strata were synthesised and screened, of which 122 (about 22%) displaced radioligand by more than 50% at 10 μM and 81 were characterised as new chemotypes in dose–response [1]. Such studies quantify how often top-ranked compounds *are* active. The complementary measurement — how a compound known in advance *not* to bind is ranked — is the one made here.

For pharmacological context, the parent alkaloid rhynchophylline has a reported functional NMDA IC_{50} of 43.2 μM in *Xenopus* oocytes [10]. That measurement concerns functional current inhibition by a different compound and does not predict the radioligand-binding behaviour of TFE-Rhy at any of the five sites. We therefore treat TFE-Rhy purely as a probe of whether the scoring pipeline over-predicts this chemotype, and draw no inference about the scaffold's pharmacology from it.

Supplementary Table S3. Assay-site classification of the ChEMBL comparison sets. Every activity record underlying the docked actives of each target, assigned to exactly one of four mutually exclusive classes by the radioligand or probe named in its `assay_description`. The denominator is the records from ChEMBL binding assays (`assay_type` = "B") for the docked actives, i.e. the records that actually entered the active sets.

Target	ChEMBL target	Records	Assays	Docked pocket	Other site	Functional	Unclassified	Organism
SERT	CHEMBL228	1,327	269	56.7%	0%	0%	43.3%	100% *H. sapiens*
5-HT_{1A}	CHEMBL214	1,499	344	72.9%	0%	0.7%	26.4%	100% *H. sapiens*
σ_1	CHEMBL287	1,517	291	52.9%	16.3%	0%	30.8%	100% *H. sapiens*
**$GABA_A$**	**CHEMBL1962**	**706**	**33**	**5.0%**	**0%**	**84.8%**	**10.2%**	100% *H. sapiens*
NMDA (pore)	assay-selected pore set	281	54	92.9%	0%	0%	7.1%	67.8% *R. norvegicus*, 3.3% *H. sapiens*

Classification is by regular-expression matching on `assay_description`; *the expressions, the per-assay assignments and the full record listing are deposited. "Unclassified" records name no diagnostic probe and are therefore not evidence of a mismatch; "other site" records name a probe for a structurally distinct site of the same protein. NMDA was site-filtered at the assay level before docking, which is why its docked-pocket fraction is high by construction. $GABA_A$ is the only target whose comparison set is dominated by a format other than radioligand binding, and the consequences are followed up in Sections 2.1 and 2.9.3.*

This classification supersedes the looser keyword audit reported at submission, which counted a wider set of records (no `assay_type` *restriction) against target-specific radioligand patterns only and gave 63.9%, 77.6% and 74.5% "assay-matched" for SERT, 5-HT_{1A} and σ_1 and 11.8% benzodiazepine-site for $GABA_A$. The two are not comparable: the denominators and the class definitions differ. All statements in this paper use the classification tabulated above.*

Supplementary Table S4. $GABA_A$ ranking by assay-defined subset of the same docked actives. Within-actives Spearman ρ between CNNaffinity and experimental potency, recomputed on subsets of the 273 docked $GABA_A$ actives defined by the assay type of their

ChEMBL records. No re-docking was performed; the subsets restrict which compounds enter the correlation.

Subset	n	ρ (raw)	permutation p	bootstrap 95% CI	ρ (RDAS)	pChEMBL IQR
All $GABA_A$ actives	273	−0.268	0.0002	[−0.382, −0.150]	−0.108	0.67
≥ 1 benzodiazepine-site record	33	**−0.304**	0.085	[−0.634, +0.059]	−0.266	0.88
Radioligand binding, no electrophysiology	74	**−0.274**	**0.018**	[−0.490, −0.048]	−0.222	1.61
Electrophysiology only	190	−0.123	0.089	[−0.261, +0.023]	+0.012	0.50

The anti-correlation is preserved, and numerically strongest, in the subsets best matched to the docked benzodiazepine site and to the radioligand-binding format; the electrophysiology-only subset gives the weakest correlation. Small-sample subsets are reported with their intervals and are not interpreted as significant on their own.

Supplementary Table S5. Ranking ρ and experimental-data quality, by target.

Target	n_act	ρ raw	ρ RDAS	% in pChEMBL [7,9]	pChEMBL IQR	% single-meas.	ρ Ki-only (n)	ρ n_meas ≥2 (n)	ρ Ki & n≥2 (n)
SERT	793	+0.020	+0.052	92%	0.96	84%	+0.058 (420)	+0.121 (123)	+0.147 (60)
NMDA	194	+0.091	−0.034	100%	0.68	87%	+0.169 (146)	−0.106 (25)	— (13)†
$GABA_A$	273	−0.268	−0.108	95%	0.67	94%	−0.184 (238)	— (16)†	— (5)†
$5\text{-}HT_{1A}$	799	+0.050	+0.011	87%	1.09	82%	+0.022 (726)	+0.191 (141)	+0.114 (117)
σ_1	792	−0.102	−0.009	93%	1.02	77%	−0.104 (633)	+0.011 (186)	+0.028 (126)

†Subset n < 20; ρ not reported / unstable. Spearman ρ over actives, score versus experimental potency.

Supplementary Table S6. AutoDock Vina sensitivity — within-active ranking.

Target	n	ρ Vina raw	ρ Vina RDAS	95% CI (raw)	ρ CNNaffinity raw

Target	n	ρ Vina raw	ρ Vina RDAS	95% CI (raw)	ρ CNNaffinity raw
SERT	793	−0.072	−0.035	[−0.138, −0.003]	+0.020
NMDA (pore)	194	−0.234	−0.245	[−0.367, −0.097]	+0.091
$GABA_A$	273	−0.101	−0.104	[−0.235, +0.029]	−0.268
$5\text{-}HT_{1A}$	799	+0.090	+0.015	[+0.023, +0.158]	+0.050
σ_1	792	−0.004	+0.025	[−0.076, +0.069]	−0.102

Vina scores negated so that higher values indicate better predicted affinity before correlation. Bootstrap 95% CI over 2,000 resamples of actives.

Supplementary Table S7. Enrichment metrics (actives vs held-out seven-descriptor-matched decoys), raw vs RDAS. Mean [2.5–97.5 percentile] over 200 independent random normalisation/evaluation partitions.

Target	Score	ROC-AUC	PR-AUC	EF1%	EF5%	EF10 %	BEDROC (α=20)	EF1% ceiling
SERT	raw	0.658 [0.643, 0.670]	0.637 [0.620, 0.658]	0.93	1.40	1.38	0.687 [0.644, 0.735]	1.94
SERT	RDAS	0.680 [0.664, 0.694]	0.669 [0.647, 0.694]	1.44	1.44	1.44	0.737 [0.686, 0.790]	1.94
NMDA (pore)	raw	0.569 [0.548, 0.591]	0.304 [0.292, 0.319]	0.00	0.17	0.78	0.165 [0.132, 0.209]	3.45
NMDA (pore)	RDAS	0.613 [0.596, 0.631]	0.358 [0.339, 0.380]	0.05	0.92	1.33	0.316 [0.245, 0.404]	3.45
$GABA_A$	raw	0.730 [0.708, 0.752]	0.551 [0.521, 0.582]	1.85	1.10	1.13	0.485 [0.431, 0.551]	2.56
$GABA_A$	RDAS	0.742 [0.721, 0.770]	0.588 [0.556, 0.623]	1.55	1.68	1.65	0.633 [0.564, 0.695]	2.56
$5\text{-}HT_{1A}$	raw	0.570 [0.556, 0.585]	0.572 [0.554, 0.589]	1.17	1.31	1.26	0.638 [0.597, 0.683]	1.98

Target	Score	ROC-AUC	PR-AUC	EF1%	EF5%	EF10 %	BEDROC (α=20)	EF1% ceiling
5-HT_{1A}	RDAS	0.567 [0.553, 0.582]	0.569 [0.554, 0.587]	1.36	1.29	1.24	0.635 [0.596, 0.679]	1.98
σ_1	raw	0.599 [0.587, 0.616]	0.620 [0.604, 0.636]	1.38	1.37	1.20	0.685 [0.648, 0.725]	1.87
σ_1	RDAS	0.614 [0.602, 0.629]	0.633 [0.618, 0.650]	1.41	1.30	1.31	0.699 [0.664, 0.743]	1.87

Enrichment-factor values are means over partitions. Within-active ranking ρ is reported in Table 2 and is computed against the full matched-decoy reference set, not per partition.

Supplementary Table S8. Multivariate null correction: ranking and enrichment by method. Mean [2.5–97.5 percentile] over 200 partitions; ρ is the partition-averaged within-active Spearman correlation for the corresponding corrected score.

Target	Method	ρ (rank)	ROC-AUC	BEDROC (α=20)
SERT	raw	+0.020	0.658 [0.643, 0.670]	0.687 [0.644, 0.735]
SERT	RDAS (MW-bin)	+0.051	0.680 [0.664, 0.694]	0.737 [0.686, 0.790]
SERT	Linear (MW+cLogP+TPSA)	+0.072	0.682 [0.668, 0.694]	0.807 [0.776, 0.839]
SERT	Random forest (6 desc)	+0.042	0.685 [0.673, 0.700]	0.782 [0.746, 0.821]
NMDA (pore)	raw	+0.091	0.569 [0.548, 0.591]	0.165 [0.132, 0.209]
NMDA (pore)	RDAS (MW-bin)	−0.027	0.613 [0.596, 0.631]	0.316 [0.245, 0.404]
NMDA (pore)	Linear (MW+cLogP+TPSA)	+0.012	0.619 [0.601, 0.637]	0.320 [0.271, 0.377]
NMDA (pore)	Random forest (6 desc)	+0.058	0.627 [0.602, 0.650]	0.382 [0.295, 0.475]
$GABA_A$	raw	−0.268	0.730 [0.708, 0.752]	0.485 [0.431, 0.551]
$GABA_A$	RDAS (MW-bin)	−0.121	0.742 [0.721, 0.770]	0.633 [0.564, 0.695]
$GABA_A$	Linear (MW+cLogP+TPSA)	−0.048	0.776 [0.757, 0.799]	0.596 [0.502, 0.667]

Target	Method	ρ (rank)	ROC-AUC	BEDROC (α=20)
$GABA_A$	Random forest (6 desc)	−0.117	0.772 [0.750, 0.798]	0.679 [0.594, 0.768]
5-HT_{1A}	raw	+0.050	0.570 [0.556, 0.585]	0.638 [0.597, 0.683]
5-HT_{1A}	RDAS (MW-bin)	+0.011	0.567 [0.553, 0.582]	0.635 [0.596, 0.679]
5-HT_{1A}	Linear (MW+cLogP+TPSA)	+0.045	0.574 [0.559, 0.591]	0.668 [0.628, 0.714]
5-HT_{1A}	Random forest (6 desc)	+0.032	0.570 [0.555, 0.585]	0.656 [0.612, 0.708]
σ_1	raw	−0.102	0.599 [0.587, 0.616]	0.685 [0.648, 0.725]
σ_1	RDAS (MW-bin)	−0.015	0.614 [0.602, 0.629]	0.699 [0.664, 0.743]
σ_1	Linear (MW+cLogP+TPSA)	−0.008	0.623 [0.609, 0.638]	0.717 [0.681, 0.763]
σ_1	Random forest (6 desc)	+0.019	0.622 [0.607, 0.636]	0.726 [0.673, 0.788]

Descriptor bias (standardised |coefficient| of CNNaffinity ~ descriptors, pooled decoys): MW 0.342 · cLogP 0.161 · TPSA 0.083 · HBA 0.045 · HBD 0.038 · rotatable bonds 0.022.

Supplementary Table S9. Both decoy generations under the identical held-out protocol.

Target	Decoys	n	AUC raw	AUC RDAS	ΔAUC	95% split interval
SERT	MW-binned	1,499	0.599	0.702	+0.103	[+0.086, +0.120]
SERT	7-descriptor	1,493	0.658	0.680	+0.022	[+0.015, +0.031]
NMDA (pore)	MW-binned	1,377	0.472	0.625	+0.153	[+0.128, +0.179]
NMDA (pore)	7-descriptor	950	0.569	0.613	+0.045	[+0.026, +0.063]
$GABA_A$	MW-binned	1,050	0.734	0.756	+0.022	[−0.003, +0.047]
$GABA_A$	7-descriptor	855	0.730	0.742	+0.013	[−0.004, +0.027]
5-HT_{1A}	MW-binned	1,500	0.580	0.561	−0.019	[−0.038, −0.000]

Target	Decoys	n	AUC raw	AUC RDAS	ΔAUC	95% split interval
5-HT_{1A}	7-descriptor	1,563	0.570	0.567	−0.003	[−0.009, +0.002]
σ_1	MW-binned	1,349	0.602	0.658	+0.055	[+0.030, +0.081]
σ_1	7-descriptor	1,380	0.599	0.614	+0.014	[+0.003, +0.024]

Supplementary Table S10. Actives versus experimentally labelled ChEMBL inactives (CNNaffinity).

Target	n_active	n_inactive	AUC raw	AUC RDAS	95% CI (raw)	Evaluable (n ≥ 50)
SERT	793	124	0.675	0.673	[0.621, 0.725]	yes
NMDA (pore)	194	210	0.739	0.704	[0.691, 0.782]	yes
$GABA_A$	273	2	0.383	0.478	[0.033, 0.755]	no
5-HT_{1A}	799	96	0.647	0.631	[0.591, 0.703]	yes
σ_1	792	62	0.624	0.594	[0.541, 0.705]	yes

Supplementary Table S11. Score-head comparison (CNNaffinity vs CNNscore vs CNN_VS). Actives versus held-out seven-descriptor-matched decoys; mean [95% split interval] over 200 partitions.

Target	Score	Corr.	ROC-AUC	BEDROC (α=20)	EF1%	EF5%
SERT	CNNaffinity	raw	0.658 [0.643,0.670]	0.687 [0.644,0.735]	0.93	1.40
SERT	CNNaffinity	RDAS	0.680 [0.664,0.694]	0.737 [0.686,0.790]	1.44	1.44
SERT	CNNscore	raw	0.685 [0.675,0.696]	0.937 [0.925,0.947]	1.89	1.93
SERT	CNNscore	RDAS	0.701 [0.686,0.714]	0.782 [0.728,0.838]	1.36	1.48
SERT	CNN_VS	raw	0.694 [0.682,0.704]	0.877 [0.849,0.904]	1.77	1.73
SERT	CNN_VS	RDAS	0.704 [0.691,0.717]	0.801 [0.741,0.855]	1.54	1.55

Target	Score	Corr.	ROC-AUC	BEDROC (α=20)	EF1%	EF5%
NMDA (pore)	CNNaffinity	raw	0.569 [0.548,0.591]	0.165 [0.132,0.209]	0.00	0.17
NMDA (pore)	CNNaffinity	RDAS	0.613 [0.596,0.631]	0.316 [0.245,0.404]	0.05	0.92
NMDA (pore)	CNNscore	raw	0.536 [0.518,0.555]	0.261 [0.217,0.302]	0.86	0.70
NMDA (pore)	CNNscore	RDAS	0.528 [0.508,0.550]	0.181 [0.146,0.222]	0.07	0.51
NMDA (pore)	CNN_VS	raw	0.554 [0.537,0.571]	0.222 [0.186,0.267]	0.33	0.64
NMDA (pore)	CNN_VS	RDAS	0.556 [0.538,0.576]	0.226 [0.181,0.294]	0.05	0.62
$GABA_A$	CNNaffinity	raw	0.730 [0.708,0.752]	0.485 [0.431,0.551]	1.85	1.10
$GABA_A$	CNNaffinity	RDAS	0.742 [0.721,0.770]	0.633 [0.564,0.695]	1.55	1.68
$GABA_A$	CNNscore	raw	0.627 [0.605,0.650]	0.467 [0.420,0.513]	1.61	1.31
$GABA_A$	CNNscore	RDAS	0.646 [0.624,0.670]	0.490 [0.417,0.561]	1.28	1.29
$GABA_A$	CNN_VS	raw	0.653 [0.631,0.675]	0.544 [0.493,0.605]	1.61	1.40
$GABA_A$	CNN_VS	RDAS	0.669 [0.648,0.691]	0.563 [0.493,0.628]	1.58	1.51
5-HT_{1A}	CNNaffinity	raw	0.570 [0.556,0.585]	0.638 [0.597,0.683]	1.17	1.31
5-HT_{1A}	CNNaffinity	RDAS	0.567 [0.553,0.582]	0.635 [0.596,0.679]	1.36	1.29
5-HT_{1A}	CNNscore	raw	0.460 [0.446,0.476]	0.450 [0.422,0.479]	1.28	0.90
5-HT_{1A}	CNNscore	RDAS	0.469 [0.454,0.485]	0.478 [0.446,0.511]	1.27	0.96
5-HT_{1A}	CNN_VS	raw	0.476 [0.461,0.491]	0.503 [0.474,0.533]	1.17	1.04
5-HT_{1A}	CNN_VS	RDAS	0.483 [0.469,0.499]	0.529 [0.497,0.562]	1.38	1.13
σ_1	CNNaffinity	raw	0.599 [0.587,0.616]	0.685 [0.648,0.725]	1.38	1.37
σ_1	CNNaffinity	RDAS	0.614	0.699	1.41	1.30

Target	Score	Corr.	ROC-AUC	BEDROC (α=20)	EF1%	EF5%
			[0.602,0.629]	[0.664,0.743]		
σ_1	CNNscore	raw	0.551 [0.531,0.566]	0.597 [0.562,0.635]	1.34	1.13
σ_1	CNNscore	RDAS	0.551 [0.535,0.565]	0.637 [0.607,0.674]	1.38	1.23
σ_1	CNN_VS	raw	0.568 [0.550,0.582]	0.665 [0.633,0.699]	1.54	1.36
σ_1	CNN_VS	RDAS	0.565 [0.549,0.579]	0.663 [0.623,0.700]	1.52	1.28

Within-active ranking (Spearman ρ) for the same score heads: none of the thirty score × correction × target combinations reached ρ ≥ 0.10; largest absolute value for CNNscore/CNN_VS was −0.175 ($GABA_A$). TFE-Rhy within-actives percentiles under CNN_VS: SERT 13.7, NMDA 6.7, $GABA_A$ 81.7, 5-HT_{1A} 70.3, σ_1 0.0 (raw).

Supplementary Table S12. Curation provenance. Record counts at each curation stage for every target, together with the number of records carrying each `standard_relation`, a `data_validity_comment`, or a `potential_duplicate` flag, and the number of distinct assays and documents contributing to each active set. All records entering the sets carried `standard_relation` "=". The largest single assay contributes 32–43 records at SERT, 5-HT_{1A}, σ_1 and NMDA, but 195 of 295 at $GABA_A$ (assay CHEMBL5737147; see Table S18). Generated by `curation_and_attrition.py`; deposited as `outputs/curation_provenance.csv`.

Supplementary Table S13. Strict-curation sensitivity. Within-active Spearman ρ and ΔAUC recomputed after re-deriving per-compound potency from records restricted to `standard_relation` "=", carrying no `data_validity_comment` and not flagged `potential_duplicate`, and after dropping compounds whose strict median pChEMBL falls below 7. Two compounds are removed in total (both 5-HT_{1A}); ρ changes by at most 0.007 and ΔAUC is unchanged at every target. Generated by `strict_curation_sensitivity.py`; deposited as `outputs/strict_curation_sensitivity.csv`.

Supplementary Table S14. Bemis–Murcko scaffold composition and scaffold-aware resampling. Per target: the number of standard and generic scaffolds, the share of molecules in singleton scaffolds, in the largest family and in the top five families, the Shannon entropy of the family-size distribution and the effective number of scaffolds exp(H); and, for ρ and ΔAUC, the conventional molecule-level bootstrap interval beside the scaffold-level cluster-bootstrap interval with the ratio of their widths. Generated by `scaffold_audit.py`; deposited as `outputs/scaffold_composition.csv` and `outputs/scaffold_bootstrap.csv`.

Supplementary Table S15. Early-recognition metrics at a matched decoy:active ratio. EF at the top 1% and 10%, BEDROC (α = 20) and PR-AUC for the site-matched subsets, computed after down-sampling the evaluation decoys to each target's original decoy:active ratio and averaging over 200 draws, with 95% intervals. Values for the full active sets are shown alongside

for comparison. Generated by `site_matched_early_enrichment.py`; deposited as `outputs/site_matched_early_enrichment.csv`.

Supplementary Table S16. Ligand-only control and nearest-neighbour diagnostic. ROC-AUC, PR-AUC and balanced accuracy for every combination of feature set (physicochemical descriptors, ECFP4, combined) and classifier (logistic regression, random forest) under scaffold-grouped five-fold cross-validation; and, for each target, the median, 95th percentile and maximum Tanimoto similarity of each decoy to its nearest active, the median similarity of each active to its nearest other active, and the percentage of actives having an analogue at Tanimoto ≥ 0.35. Standardised mean differences for the seven matching descriptors are reported in the same table. Generated by `ligand_only_control.py`, `decoy_separability_diagnostic.py` and `decoy_generation_guard.py`. Deposited as outputs/ligand_only_control.csv and outputs/decoy_separability.csv.

Supplementary Table S17. Site-matched enrichment on rebuilt decoys. Assay classification counts per target and condition; and, for the full active set and the site-matched subset, the number of actives and decoys, raw and corrected AUC, ΔAUC with both the 200-partition split interval and the compound-level bootstrap interval, and the within-active ρ. The site-matched rows use decoys rebuilt against the subset and newly docked where required. Generated by `site_matched_benchmark.py`, `match_decoys_subsets.py` and `site_matched_enrichment_v2.py`; deposited as `outputs/assay_site_classification.csv` and `outputs/site_matched_enrichment_v2.csv`.

Supplementary Table S18. $GABA_A$ source heterogeneity. Within-active ρ with bootstrap interval, permutation p-value, and the means of CNNaffinity and pChEMBL, for the pooled active set, for the 190 molecules whose potency comes from assay CHEMBL5737147, and for the remaining 83; the pooled estimate after rank-centring within source; and the leave-one-out influence range. Generated by `gaba_source_heterogeneity.py`; deposited as `outputs/gaba_source_heterogeneity.csv`.

Supplementary Table S19. Cognate-ligand redocking, convergence and pose reranking. Per target and cognate ligand: median, interquartile range and full range of top-1 RMSD over fifty explicit seeds, the fraction of seeds within 2 Å, the same quantities for the best of nine poses, median contact recovery and centroid displacement, and the CNNaffinity of the crystallographic pose beside the median CNNaffinity of the production-selected poses. A convergence table reports each estimate as a function of the number of seeds used, from three to fifty. A third panel reports, per seed, the RMSD of the Vina rank-1 pose, the RMSD of the pose GNINA ranks first when the complete nine-pose list is rescored, the best RMSD available in the list, and the rank of that best pose under each scoring order.

Major binding interactions recovered, per target, out of the three closest crystallographic contacts (full detail in Table S23): SERT **3 of 3**, σ_1 **3 of 3**, 5-HT_{1A} **3 of 3**, NMDA **2 of 3**, $GABA_A$ **1 of 3**, where an interaction counts as recovered if the top-ranked redocked pose re-forms it in a majority of the fifty seeds. Note that this does not track RMSD: σ_1 and 5-HT_{1A} recover all three principal interactions despite median top-1 RMSDs of 3.31 Å and 8.74 Å.

Generated by redock_cognate.py, rerank_poses.py and interaction_recovery.py; deposited as outputs/redock_cognate.csv, outputs/redock_cognate_per_seed.csv, outputs/redock_convergence.csv and outputs/rerank_poses.csv.

Supplementary Table S20. Compound attrition. For every target: the number of molecules with a pChEMBL value, the actives and inactives surviving the activity thresholds and the 800-compound cap, the number docked, the number with a usable CNN score, the number lost in ligand preparation or docking, the number of property-matched decoys selected, and the number of decoys with a usable CNN score. Generated by curation_and_attrition.py; deposited as outputs/attrition_table.csv.

Supplementary Table S21. Docking stochasticity. For each target: the median and maximum spread of per-molecule CNNaffinity across five explicit seeds, the median between-seed rank correlation, the mean, range and standard deviation of raw enrichment AUC and of within-active ρ across seeds, and the corresponding values computed from the archived production scores for the identical sampled compounds. Generated by seed_stability.py and seed_stability_analysis.py; deposited as outputs/seed_stability.csv and outputs/seed_stability_summary.csv.

Supplementary Table S22. ChEMBL assay–target confidence. For every assay contributing to the five active sets: ChEMBL's confidence score (0–9, where 9 denotes a direct single-protein target assignment), summarised per target as the score distribution, median, minimum, and the percentage of contributing records at 8 or above. SERT, $GABA_A$, 5-HT_{1A} and σ_1 draw 100% of their records from assays scored 8 or 9; NMDA has a median of 4 and a minimum of 0, reflecting its assembly from channel-blocker binding assays on rodent brain homogenate. Generated by assay_confidence_audit.py; deposited as data/assay_confidence.csv and outputs/assay_confidence_summary.csv.

Supplementary Table S23. Preservation of the major binding interactions on redocking. For each target, the three residues making the closest heavy-atom contacts with the co-crystallised ligand, the geometric class of each contact (salt bridge or charged hydrogen bond where a charged residue's N/O lies within 3.5 Å of a ligand N/O; hydrogen bond or polar contact for any other N/O pair within 3.5 Å; aromatic residue contact for Phe, Tyr, Trp and His; otherwise hydrophobic contact), the contact distance in the crystal structure, and the fraction of the fifty redocking seeds in which the top-ranked pose re-forms that contact within 4 Å. Residues are identified from the deposited coordinates rather than from the literature, and are reported with their chain and residue number as they appear in the prepared receptor. Generated by interaction_recovery.py; deposited as outputs/interaction_recovery.csv.
